# Superoxide Anion-Dependent Mitochondrial Fission Contributes to Hippocampal Synaptic Dysfunction in Stress-Susceptible Mice

**DOI:** 10.1021/jacsau.5c00493

**Published:** 2025-09-15

**Authors:** Xiwei Li, Xue Xue, Simiao Zhang, Tony D. James, Ping Li, Xin Wang, Bo Tang

**Affiliations:** † College of Chemistry, Chemical Engineering and Materials Science, Key Laboratory of Molecular and Nano Probes, Ministry of Education, Collaborative Innovation Center of Functionalized Probes for Chemical Imaging in Universities of Shandong, Institutes of Biomedical Sciences, 47856 Shandong Normal University, Jinan 250014, P. R. China; ‡ College of Chemistry and Chemical Engineering, Northwest Normal University, Lanzhou, Gansu Province 730070, People’s Republic China; § Laoshan Laboratory, 168 Wenhai Middle Rd Aoshanwei Jimo, Qingdao, Shandong 266237, China; ∥ Department of Chemistry, University of Bath, Bath, Somerset BA2 7AY, United Kingdom; ⊥ School of Chemistry and Chemical Engineering, Henan Normal University, Xinxiang, Henan 453007, P. R. China

**Keywords:** fluorescence imaging, superoxide anion, depression, mitochondrial fission

## Abstract

Reduced synaptic plasticity of hippocampal neurons is a core aspect of depression. Mitochondrial dysfunction affects the synaptic plasticity of neurons. However, the characteristics and molecular mechanisms of mitochondrial dysfunction in hippocampal neurons remain unclear. Oxidative stress observed in depression suggests that excess superoxide anion radical (O_2_^•–^), the first ROS generated in mitochondria, may play crucial roles in mediating mitochondrial damage associated with depression. Unfortunately, current small-molecule fluorescent probes may suffer from diffusion after reacting with O_2_^•–^, thereby limiting the accuracy of studying O_2_^•–^’s biological roles in subcellular structures. Thus, we exploited a fluorescence sensing and labeling strategy for accurately acquiring spatiotemporal information on O_2_^•–^. The fluorescent probe (RB-FM) could react with O_2_^•–^, triggering the generation of a covalent fluorescent label that binds to nearby biological nucleophiles. This action facilitates high-precision *in situ* imaging of O_2_^•–^ during mitochondrial dysfunction. The imaging results demonstrated a reduction in dendritic spine density in hippocampal neurons of stress-susceptible mice, accompanied by a significant increase in mitochondrial O_2_^•–^ (mtO_2_^•–^)-dependent mitochondrial peripheral fission. Notably, we found an intriguing form of mitochondrial damage: mitochondrial peripheral fission increased, while total mitochondrial fission and mitophagy were unaffected. We further identified a depression-associated pathological cascade beginning with elevated Ca^2+^ levels in hippocampal neurons, which triggers mtO_2_^•–^-dependent reductions in Coq4 and elevations in Parkin, driving mitochondrial peripheral fission and reducing synaptic plasticity. This work provides a mechanistic framework for O_2_^•–^ control of mitochondrial peripheral fission and demonstrates how redox signaling relates to synaptic plasticity in depression.

## Graphical abstract

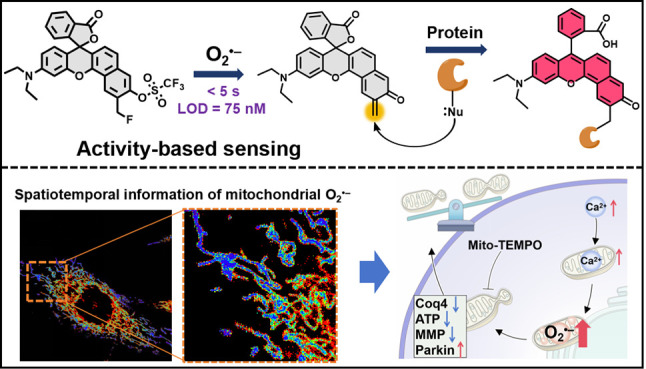

## Introduction

Major depressive disorder (MDD), a principal cause of disability worldwide, leads to heavy economic and social burdens, which highlight the need to delve into the specific molecular mechanisms of depression.^1^ Altered brain structure and function are among the most common neurobiological characteristics of MDD.^2,3^ Neuroimaging studies and autopsy reports have confirmed reductions in hippocampal volume and synaptic plasticity in patients with MDD.^4–6^ By generating energy (ATP and NAD^+^) and regulating redox signaling, mitochondria play significant roles in fundamental synaptic activity and plasticity.^7–10^ Unsurprisingly, mitochondrial damage can lead to reduced synaptic plasticity and contribute to depression. However, the characteristics and molecular mechanisms of mitochondrial damage in the hippocampus in depression are not yet clear.

Oxidative stress in the brain is a central feature of depression.^11,12^ Excess reactive oxygen species (ROS) can lead to mitochondrial oxidative imbalance and dysfunction.^13,14^ The levels of O_2_^•–^, the first ROS produced by mitochondria, directly reflect the degree of oxidative stress in mitochondria.^15^ Considering excess O_2_^•–^ could interact with a variety of proteins and cause mitochondrial damage, we hypothesize that elevated mitochondrial O_2_^•–^ (mtO_2_^•–^) levels might cause mitochondrial dysfunction in the hippocampus of stress-susceptible mice.^16^ Therefore, to understand the molecular mechanism of depression, it is imperative to precisely quantify the changes of mtO_2_^•–^ *in situ* within the hippocampus of mice with depressive-like behavior. Furthermore, elucidating the dynamic spatial and temporal fluctuations of O_2_^•–^ will shed light on the role of O_2_^•–^ during mitochondrial damage and morphological changes observed in the hippocampus of stress-susceptible mice.

Fluorescence imaging has increasingly emerged as a pivotal tool in biomedical research, owing to its exceptional selectivity, heightened sensitivity, and capability for *in situ* imaging.^17^ Our group has pioneered a reversible fluorescent probe based on phenol-quinone interconversion and an ultrasensitive fluorescent probe based on chemiluminescence resonance energy transfer for O_2_^•–^ detection.^18,19^ However, native intracellular environments are heterogeneous, and the biological functions of active molecules are closely tied to their spatial locations.^20,21^ Especially, the low concentration and high reactivity of O_2_^•–^ lead to more localized responses.^22^ These make it essential to record the spatiotemporal dynamics of O_2_^•–^ to accurately reflect its flux in various subcellular regions and understand its role in pathological conditions. However, fluorescent probes that migrate after reacting with O_2_^•–^ fail to accurately convey spatial information.^23^ This issue becomes particularly acute during studies of mitochondrial damage, as MMP dissipation may cause electrophoretically targeted O_2_^•–^ probes to leak out of mitochondria. Thus, developing O_2_^•–^ fluorescent probes with spatiotemporal labeling properties is crucial for resolving the role of mtO_2_^•–^ in mitochondrial damage within the hippocampus of stress-susceptible mice.

Here, we created a fluorescent probe, RB-FM, for the accurate imaging of mitochondrial O_2_^•–^ *in situ*, which uses a sensing and labeling strategy via tandem active groups. The probe RB-FM, designed with quinone-methide chemistry, rapidly binds to the surrounding proteins and releases fluorescence upon reaction with O_2_^•–^ (Scheme 1). This allows for the detection of the O_2_^•–^ flux and localization, facilitating the accurate tracking of the spatial and temporal changes of O_2_^•–^ during mitochondrial damage. *In situ* imaging, proteomics, and transcriptomics revealed that the mitochondria in neurons of the hippocampal region in mouse brains with depression exhibit increased O_2_^•–^-dependent peripheral fission (division at the periphery that is responsible for clearing damaged mitochondrial segments via mitophagy), attributed to elevated Ca^2+^ levels.

**Scheme 1:**
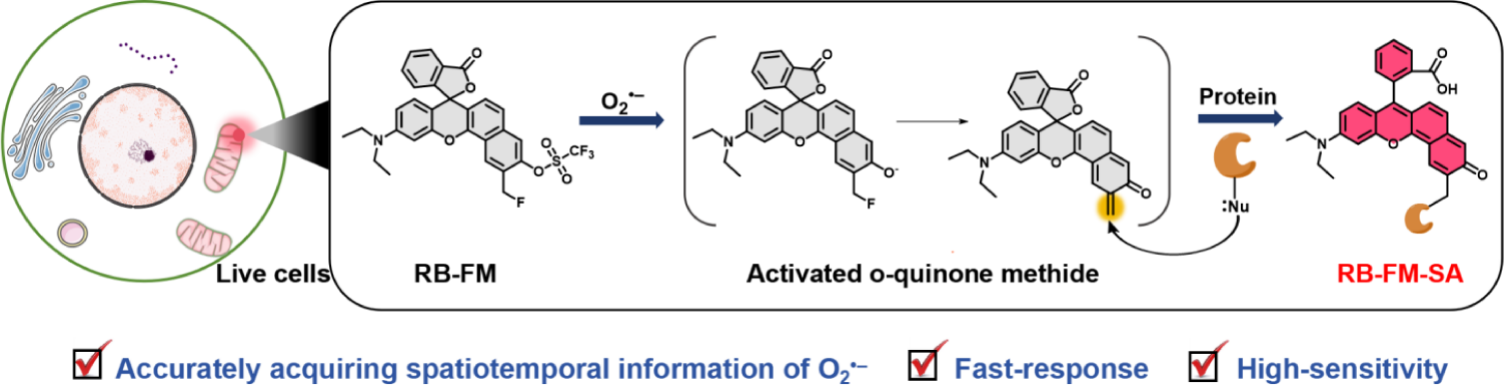
Schematic Diagram of RB-FM in Response to O_2_^•–^ Illustrates that the Activation of Fluorescence and the Binding of a Nucleophile (Nu) within the Adjacent Protein Occur Simultaneously

## Results and Discussion

### Design, Synthesis, and Optical Properties of RB-FM

To investigate whether mtO_2_^•–^ regulates mitochondrial damage, it is necessary to accurately capture the spatiotemporal variation of O_2_^•–^ during the process of mitochondrial dysfunction. Hence, we employed quinone-methide chemistry to design a self-immobilized fluorescent probe.^24–26^ This strategy uses the fact that the fluorescent product can permanently link with the protein thus counteract the diffusion of the *in situ* probe, allowing for accurate measurement of the localization of O_2_^•–^ and facilitating the precise detection of O_2_^•–^ fluxes in specific subcellular regions.^27–29^ The rapid fluctuation of mtO_2_^•–^ requires a probe that can respond instantaneously. Therefore, we chose trifluoromethylsulfonyl as the specific recognition group for O_2_^•–^.^30–32^ To meet the above requirements, dyes need to have excellent photophysical properties and multiple morphologically modifiable sites. So, seminaphthorhodafluor seems to be an appropriate choice.^33^ Upon reaction with O_2_^•–^, the conversion of trifluoromethylsulfonyl to the corresponding phenol triggers fluoride elimination, generating a highly reactive quinone methide electrophile. When formed intracellularly, this electrophile reacts with proximal protein-based nucleophiles, resulting in a fluorescent product covalently labeled at the site of the activity-based sensing response (Scheme 1). Briefly, an aldehyde group is first introduced adjacent to the hydroxyl group on the seminaphthorhodafluor. Subsequently, trifluoromethylsulfonyl is attached to the hydroxyl site. The aldehyde group is then converted to benzyl fluoride through reduction and fluorination reactions to obtain the fluorescent probe RB-FM (Figure S1). To verify the spatial labeling ability of RB-FM, we also prepared the control probe RB-SA (which lacks the benzyl fluoride structure) and the activated probe RB-FM-SA (Scheme 1). All of their structures have been characterized by HRMS and NMR spectroscopy (Figure S21-31).

After preparing the probe RB-FM, we examined its optical properties. RB-FM exhibited absorption peaks at 568 nm (Figure S2a) and fluorescence peaks at 615 nm (Figure 1a), which increased after reacting with O_2_^•–^ within 5 s (Figure 1b). Varying the O_2_^•–^ concentration from 0 to 1.0 μΜ, the dose-dependent responses of RB-FM to O_2_^•–^ exhibited a linear regression equation *F*_615 nm_ = 3898.8 [O_2_^•–^] (μM) + 14.205 with a correlation coefficient of 0.996, yielding a detection limit of 0.75 nΜ (Figure 1c,d). When the concentration of O_2_^•–^ increased continuously, the fluorescence intensity of the probe rose, gradually reaching a plateau (at ∼5 μM, Figure S2b,c). Moreover, RB-FM produced a highly selective response for O_2_^•–^ over other biologically relevant ROS, metal ions, and nucleophiles and was unaffected over a pH range from 6.5 to 9.0 (Figure S3a-c). In addition, RB-FM also possesses excellent photostability (Figure S3d). These results demonstrate the probe’s superior sensitivity and selectivity toward O_2_^•–^ (Table S1).

**Figure 1:**
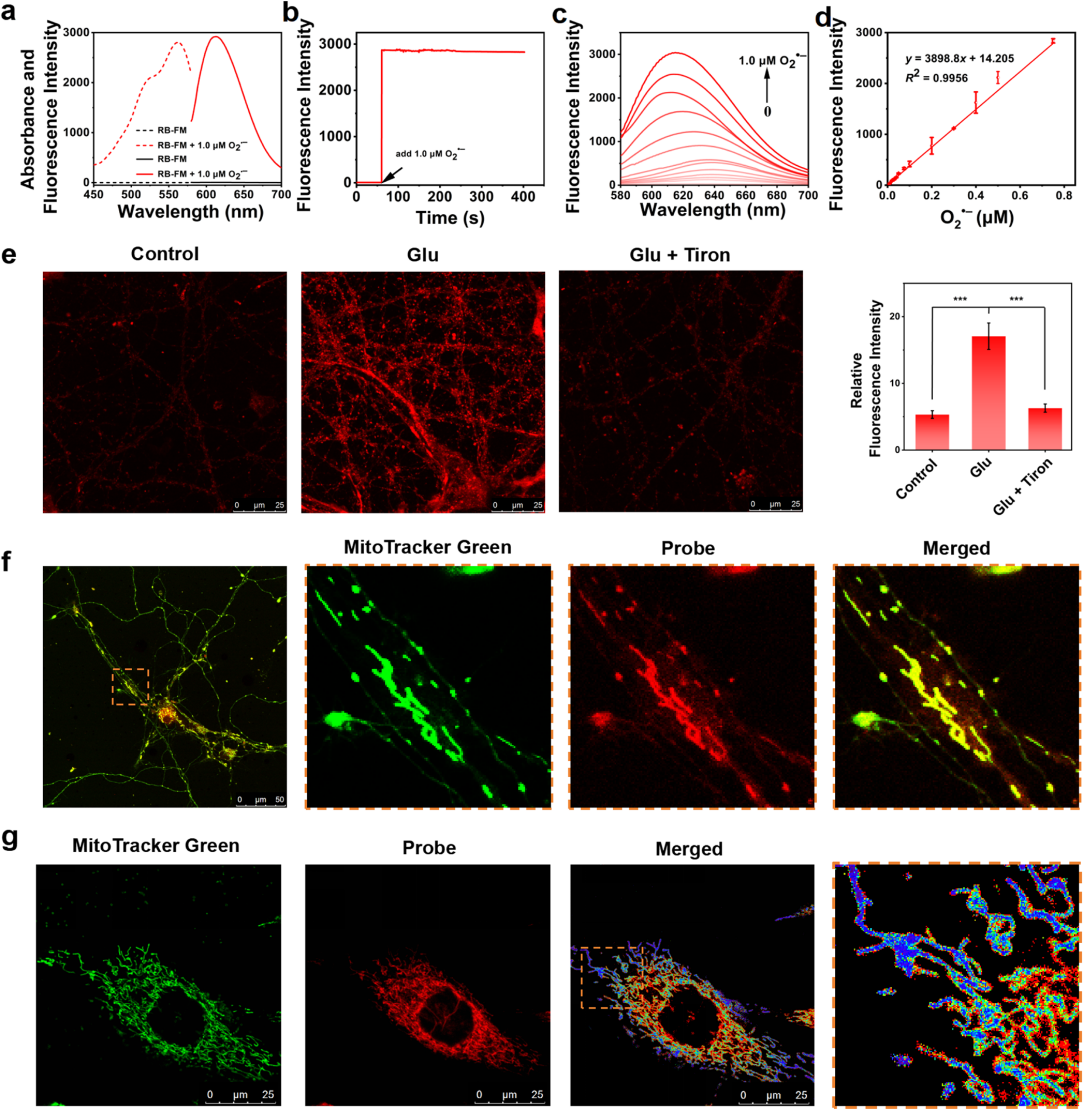
Spectral response and fluorescence imaging analysis in primary neurons of RB-FM toward O_2_^•–^. (a) Absorption spectra and fluorescence spectra of RB-FM (5.0 μM) in PBS (pH 7.4) before and after incubation with O_2_^•–^ (1.0 μM) within 1 min. (b) Fluorescence intensity changes of RB-FM before and after its reaction with O_2_^•–^ over time (0–400 s). (c) Fluorescence spectra of RB-FM (5.0 μM) after the reaction with different concentrations of O_2_^•–^ (0–1.0 μM). (d) Linear relationship between the fluorescence intensity of RB-FM (5.0 μM) at 615 nm and different concentrations of O_2_^•–^ (0–0.75 μM), λ_ex_/_em_ = 561/615 nm. (e) Confocal imaging of endogenous O_2_^•–^ in primary neurons treated with Glu (100 μM), Glu (100 μM), and Tiron (50 μM), and relative red fluorescence intensity output of various groups (λ_ex_ = 561 nm, λ_em_ = 580–680 nm). (f) Confocal imaging in primary neurons and (g) PC12 cells with RB-FM (10 μM, λ_ex_ = 561 nm, λ_em_ = 580–680 nm) and MitoTracker Green (100 nM, λ_ex_ = 488 nm, λ_em_ = 500–550 nm). Scale bar = 10 μm (f) and 5 μm (e, g).

### The Spatial Labeling Performance of RB-FM

To test the spatial labeling performance of RB-FM in an O_2_^•–^-dependent manner, we incubated RB-FM or RB-SA with bovine serum albumin (BSA) as a model protein substrate in the presence or absence of O_2_^•–^ (0–1.0 μM), and the reactions were analyzed by sodium dodecyl sulfate-polyacrylamide gel electrophoresis (SDS-PAGE). It was observed that only incubation with probe RB-FM results in an increase in the fluorescence intensity of the protein bands, whereas incubation with probe RB-SA does not (Figure S4). This indicates that the benzyl fluoride moiety in RB-FM is crucial for achieving covalent labeling of proteins in response to O_2_^•–^. To better simulate the physiological conditions of O_2_^•–^ generation in live systems, xanthine oxidase (XO) was utilized as the source of O_2_^•–^. When coincubated with xanthine and XO, RB-FM triggered fluorescent covalent labeling of XO, which was attenuated by superoxide dismutase (SOD) addition (Figure S5). Furthermore, in the presence of both XO and BSA, RB-FM selectively labeled XO (Figure S6). In addition, high-resolution LC-MS experiments were performed to confirm the proposed fluorescence sensing and labeling mechanisms. LC-MS of RB-FM (*t*_R_ = 3.461 min, [M + H]^+^, *m*/*z* = 602.1257, Figure S32) and RB-FM + O_2_^•–^ showed cleavage of RB-FM to the related phenol (*t*_R_ = 2.003 min, [M]^+^, *m*/*z* = 468.1799, Figure S33). These results collectively indicate that probe RB-FM can rapidly achieve fluorescent covalent labeling of adjacent proteins after responding to O_2_^•–^.

### RB-FM Can Acquire Spatiotemporal Information of O_2_^•–^ in the Mitochondria of Living Neurons

Before using RB-FM to image O_2_^•–^ in biological systems, its cytotoxicity was evaluated by using a CCK-8 assay. It was found that RB-FM exhibited good biocompatibility at a concentration of 50 μM, which are higher than those used for imaging (10 μM) (Figure S7). Next, we examined the ability of the probe to image O_2_^•–^ in living neurons. Previous studies suggest that glutamate (Glu) is a major endogenous excitatory neurotransmitter, facilitating normal synaptic transmission and plasticity. However, elevated concentrations of Glu can excessively stimulate glutamatergic receptors and lead to calcium influx and protein kinase C (PKC) translocation, ultimately causing an oxidative burst and oxidative stress. To initiate oxidative stress, primary neurons were pretreated with 100 μM Glu for 1 h. These neurons elicited brighter fluorescence (about 3-fold) than control neurons (Figure 1e). To further confirm that the enhancement of the probe fluorescence intensity can be ascribed to the elevated concentration of O_2_^•–^ in the neurons, we incubated the Glu-stimulated primary neurons with Tiron (a specific scavenger of O_2_^•–^) and found a significant inhibition (2.7-fold) of the fluorescence intensity compared to the primary neurons without Tiron (Figure 1e). These results indicated that the RB-FM probe could specifically image changes in the levels of O_2_^•–^ in primary neurons. Moreover, the capability of RB-FM to image endogenous O_2_^•–^ was validated in PC12 cells (Figure S8).

Interestingly, we observed that RB-FM was likely to accumulate in the mitochondria. To determine the precise location of RB-FM, we coincubated the primary neurons with MitoTracker Green and RB-FM. As shown in Figure 1f, clear mitochondrial structures were observed using 100× oil immersion objective, and the fluorescence from RB-FM exhibited a strong overlap with that of MitoTracker Green, evidenced by a Pearson’s coefficient of 0.85. This may be due to mitochondria being the primary organelles for ROS production in neurons. To further verify that probe RB-FM achieves spatiotemporal tracing of mitochondrial O_2_^•–^ through covalent protein labeling rather than mitochondrial aggregation of its activated form (RB-FM-SA) via electrostatic interactions, we performed cellular imaging with RB-FA-SA. The results showed that RB-FM-SA lacked effective mitochondrial localization (Pearson’s coefficient: 0.42, Figure S9). Furthermore, RB-SA and RB-FM exhibited distinct subcellular distributions (Figure S10). Additionally, unlike imaging with RB-SA, the fluorescence intensity of RB-FM persisted after extensive washing or upon mitochondrial membrane potential (MMP) dissipation (Figure S11). These data demonstrate that RB-FM covalently labels mitochondria in response to O_2_^•–^. Notably, unlike MitoTracker Green, which uniformly labels all mitochondria in cells, probe RB-FM exhibits heterogeneous fluorescence intensity across individual mitochondria and even within subdomains of single mitochondria (Figure 1g). These findings permitted us to obtain precise spatiotemporal information on O_2_^•–^ during mitochondrial damage.

### Elevated mtO_2_^•–^ and Mitochondrial Peripheral Fission in the Hippocampus of Stress-Susceptible Mice

We next observed spatiotemporal changes in the levels of O_2_^•–^ in the mitochondria of hippocampal neurons in depressed mice. First, C57BL/6J male mice were subjected to 28 consecutive days of chronic unpredictable mild stress (CUMS) and then separated into stress-susceptible and resilient populations (SS and SR) based on the measurement of depression-like behavior (Figure 2a,b).^34^ Subsequently, hippocampal dendritic plasticity was assessed by Golgi staining in each group of mice. As shown in Figure 2c, the density of neuronal dendritic spines in the hippocampal region was significantly reduced in mice exhibiting susceptibility to stress. This prompted us to investigate whether these changes in the hippocampus are related to mtO_2_^•–^. To assess this, we imaged mtO_2_^•–^ in the hippocampus of different groups using RB-FM. Compared with the control group, the fluorescence intensity of hippocampal slices from stress-susceptible and stress-resilient groups increased by 8-fold and 2-fold, respectively (Figure 2d). This indicates a significant elevation in mtO_2_^•–^ in the hippocampus of stress-susceptible mice. The increased mtO_2_^•–^ reflects the fact that the mitochondria are experiencing oxidative stress, which may lead to mitochondrial damage. Thus, we evaluated the mitochondrial status by examining two key indicators, MMP and ATP levels in the hippocampal mitochondria. We found that the MMP in the stress-resilient and stress-susceptible groups decreased by 14% and 20% relative to the control group, respectively. Additionally, ATP levels decreased by 10% and 60% relative to the control group, respectively (Figure 2e), indicating mitochondrial damage.

**Figure 2:**
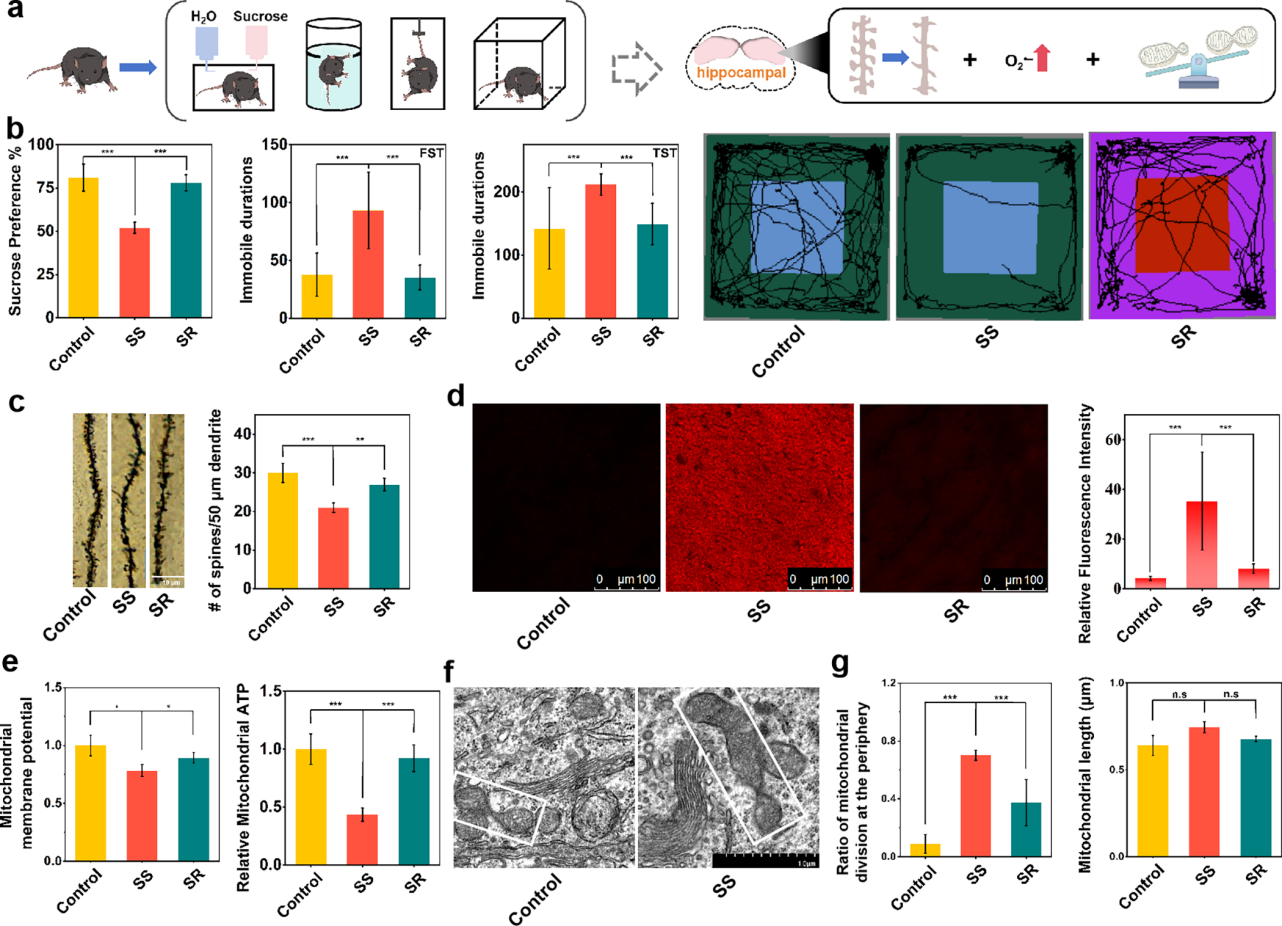
Depressive-like behavior tests of mice and alterations in dendritic spine density, O_2_^•–^ concentration, and mitochondrial state in the hippocampus. (a) Experimental diagram of depression-like behavior tests and hippocampal physiological changes in susceptible mice. (b) Sucrose preference tests, immobile durations of mice in forced swimming tests, immobile durations of mice in tail suspension tests, and open field tests. (c) Golgi staining imaging of hippocampal neurons in various groups of mice, and dendritic spine density data. (d) O_2_^•–^ imaging of hippocampal mitochondria in various groups of mice using probe RB-FM (5.0 μM, λ_ex_ = 561 nm, λ_em_ = 580–680 nm), and data output. Scale bar = 20 μm. (e) Hippocampal mitochondrial membrane potential and mice hippocampal mitochondrial ATP concentration in each group. (f) Mitochondrial fission in hippocampal neurons of control mice and stress-susceptible mice. (g) The ratio of mitochondrial peripheral fission and mitochondrial size in hippocampal neurons of each group.

Mitochondrial morphology and status are closely interconnected and can serve as indicators for determining the type of mitochondrial damage.^35^ Accordingly, we performed electron microscopic imaging of the hippocampal slices. Notably, we found the ratio of peripheral fission to the total number of mitochondrial fissions in the hippocampus of stress-resistant and stress-susceptible mice to be increased by 4.2-fold and 7.9-fold, respectively, when compared to the control group (Figure 2f,g). However, we did not observe significant differences in the total number of mitochondrial fissions in the hippocampal neurons of mice in each group. An increase in mitochondrial fission produces more small-sized mitochondria, but we did not observe changes in mitochondria size (Figure 2g), which suggests that intermediate fission, which represents mitochondrial biogenesis, is decreased in hippocampal neurons in depressed mice, whereas peripheral fission, which represents impaired mitochondrial function, is increased. Moreover, the size distribution of mitochondria in hippocampal neurons was broader, and the mitochondrial morphology varied more among the groups of mice (Figure S12). All these results support the occurrence of impaired mitochondrial function, which can be characterized by increased peripheral fission in hippocampal neurons of stress-susceptible mice. Given that elevated ROS is a hallmark precursor to mitochondrial peripheral fission and that O_2_^•–^ is the primary ROS produced by the mitochondria, it is reasonable to speculate that high levels of high-activity O_2_^•–^ may induce oxidative stress in the mitochondria. This, in turn, could lead to decreased MMP and ATP, followed by peripheral fission, ultimately contributing to a reduction in synaptic plasticity.

### Mitochondrial Peripheral Fission Exhibits O_2_^•–^ Dependence

To further investigate the relationship between O_2_^•–^ and mitochondrial fission in the hippocampus, as well as the resulting reduction in synaptic plasticity, we first examined the effects of elevated O_2_^•–^ on the mitochondria. Our findings revealed that Glu incubation led to an increase of mtO_2_^•–^and decrease in MMP. Conversely, the application of Mito-TEMPO (a mitochondria-targeted O_2_^•–^ scavenger) restores the reduction in MMP caused by Glu incubation (Figure 3a). This suggests that elevated O_2_^•–^ may lead to a decrease in MMP and subsequent mitochondrial damage. We further explored the effects of elevated O_2_^•–^ on mitochondrial morphology. Prolonged dynamic imaging with MitoTracker Deep Red revealed significant swelling and fragmentation of the mitochondria after Glu incubation as well as the emergence of mitochondrial peripheral fission. Importantly, this fragmentation was similarly mitigated by the scavenging of mtO_2_^•–^ (Figure 3b,c). To obtain more direct evidence, we dynamically observed synergistic changes in mtO_2_^•–^ and morphology in neurons using the probe RB-FM. We found that elevated levels of mtO_2_^•–^ triggered mitochondrial peripheral fission (Figure 3d,e). These results suggest that mitochondrial peripheral fission is dependent on mtO_2_^•–^. To determine whether mitochondrial peripheral fission mediated by elevated O_2_^•–^ impacts neuronal synaptic plasticity, we examined morphological changes in neuronal dendritic spines during Glu incubation. It was found that Glu incubation significantly altered dendritic spine density, whereas neurons not exposed to Glu exhibited no significant changes in dendritic spine density over the same time period. Scavenging O_2_^•–^ with Mito-TEMPO during Glu incubation also prevented the Glu-induced alterations in dendritic spine density (Figure S13). These findings suggest that high concentrations of mtO_2_^•–^ in the hippocampus of stress-susceptible mice mediated the increased level of mitochondrial peripheral fission and decreased neuronal synaptic plasticity.

**Figure 3:**
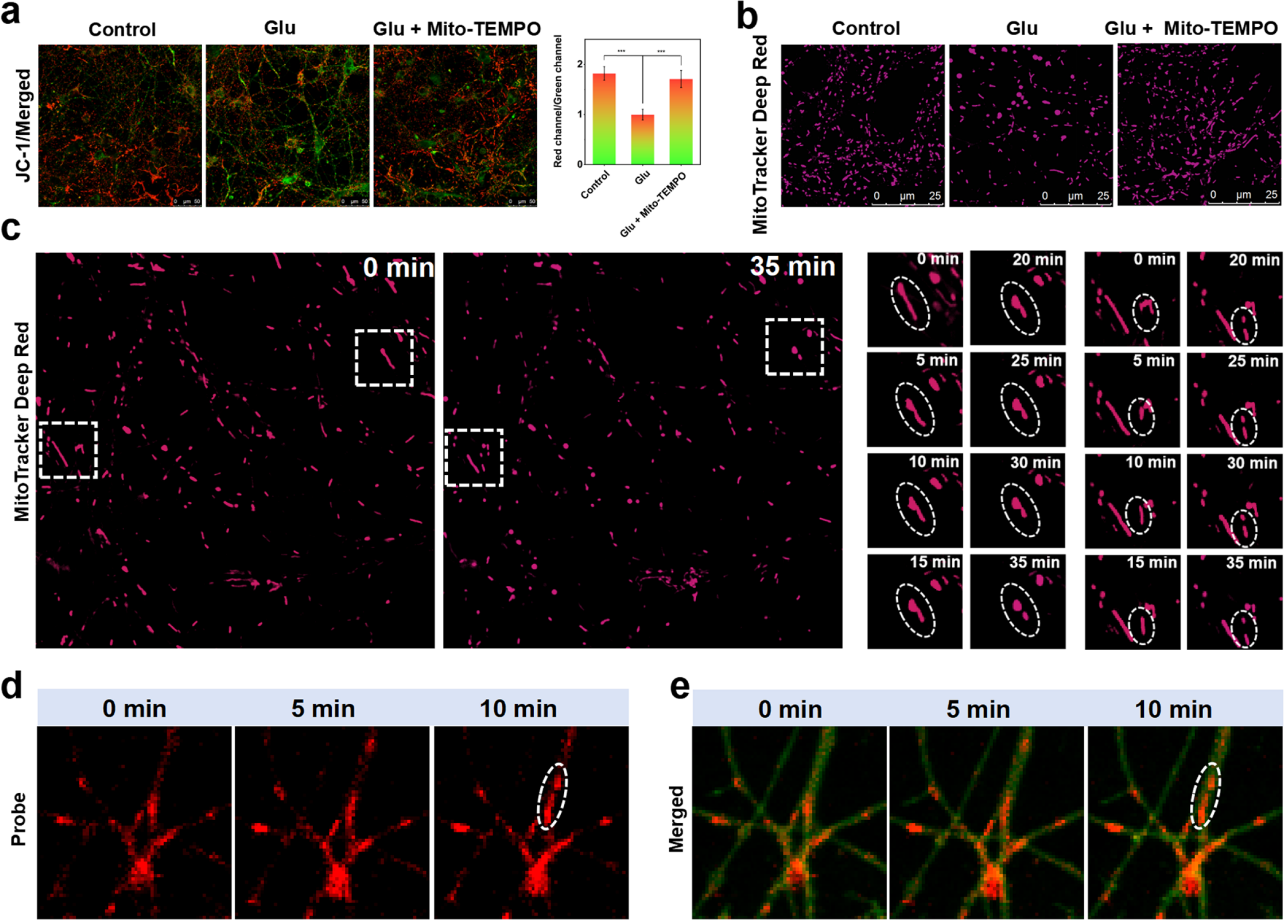
O_2_^•–^-dependent impairment of mitochondrial function and mitochondrial peripheral fission. (a) JC-1 detection for MMP of primary neurons or after incubation with Glu (100 μM) for 1 h, incubation with Glu (100 μM) and Mito-TEMPO (10 μM) for 1 h, and data output. (b) Changes in mitochondrial morphology of primary neurons, incubation with Glu (100 μM) for 1 h, and incubation with Glu (100 μM) and Mito-TEMPO (10 μM) for 1 h. (c) Confocal imaging of dynamic changes of mitochondrial morphology in primary neurons incubated with Glu, and some mitochondrial peripheral fission. (d) Confocal imaging of O_2_^•–^ in mitochondria and mitochondrial peripheral fission in primary neurons using probe RB-FM (10 μM, λ_ex_ = 561 nm, λ_em_ = 580–680 nm) and (e) overlay with Dio dye.

### Elevated mtO_2_^•–^ Mediates Mitochondrial Peripheral Fission by Reducing Mitochondrial Biogenesis and Disrupts Redox Homeostasis

To elucidate the specific molecular mechanisms of O_2_^•–^-mediated mitochondrial peripheral fission, we first reviewed the usual mechanisms of mitochondrial fission. Numerous studies have suggested that it is the ratio of dynamin-related protein 1 (Drp1) to mitofusin 2 (Mfn2) that coregulates mitochondrial fission and fusion behavior.^36–38^ To determine whether elevated O_2_^•–^ affects mitochondrial fission by influencing the expression of Drp1 and Mfn2, we examined their expression in the hippocampus. The results indicated no significant differences in the levels of Drp1 and Mfn2 across all groups of mice (Figure S14). This suggests that the increased mitochondrial peripheral fission in the hippocampus of stress-susceptible mice is not driven by O_2_^•–^-mediated changes in Drp1 and Mfn2 expression. Then, we conducted proteomic and transcriptomics analysis to identify differences in mitochondria-related proteins and transcription in the hippocampus of each group (Figure S15–18). Significantly, we also did not observe changes in optic atrophy 1­(Opa1), mitofusin 1 (Mfn1), and mitochondrial fission protein 1 (Fis1) at the protein and transcriptional levels. This result is consistent with our observation that the total number of mitochondrial fissions did not change significantly, but only the ratio of mitochondrial peripheral fission changed. In addition, we observed reduced expression levels of mitochondrially encoded ATP synthase membrane subunit 8 (Mtatp8), which are involved in ATP synthesis, as well as coenzyme Q4 (Coq4) and mitochondrial ubiquinol-cytochrome c reductase complex assembly factor 3 (Uqcc3), which help maintains mitochondrial redox homeostasis.^39–42^ Additionally, we found a decrease in mitochondrial ribosomal protein S21 (MrpsS21) and mitochondrial ribosomal protein 33 (Mrps33), ribosomal subunits responsible for the synthesis of mitochondria-associated proteins (Figure 4b). The reduction in mitochondrial biogenesis and disruption of redox homeostasis not only indicate mitochondrial functional impairment but may also trigger mitochondrial peripheral fission to eliminate damaged sites.^43^ This is consistent with the observed increase in Parkin levels (Figure 4b,c). To further confirm the relationship between these alterations and mtO_2_^•–^, we assessed the impact of elevated mtO_2_^•–^ in neurons on the expression of these proteins by a Western blot assay. We found that increasing mtO_2_^•–^ with Glu incubation dramatically promotes Parkin expression, while scavenging O_2_^•–^ in the mitochondria decreased the expression of Parkin (Figure 4d). Similarly, O_2_^•–^ negatively regulated Coq4 expression (Figure 4d). Combined with the previous evidence, these results suggest that elevated mitochondrial O_2_^•–^ mediates the onset of mitochondrial peripheral fission by reducing mitochondrial biogenesis and disrupting redox homeostasis.

**Figure 4:**
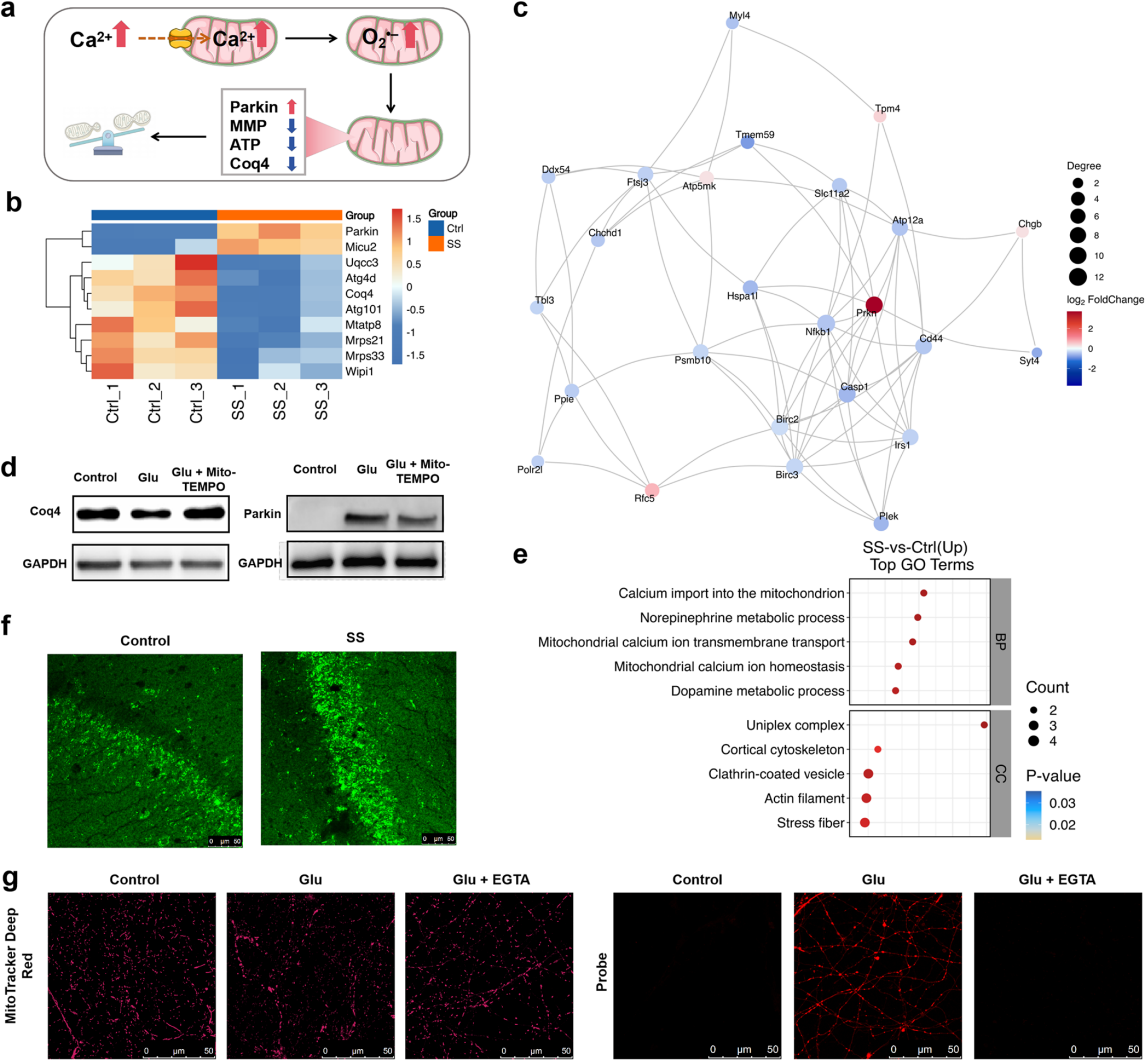
Elevated hippocampal Ca^2+^ concentration in the stress-susceptible mice leads to increased mtO_2_^•–^, driving mitochondrial peripheral fission. (a) Experimental diagram of the pathological cascade: elevated Ca^2+^ levels in hippocampal neurons, which triggers mtO_2_^•–^-dependent reductions in Coq4 and elevations in Parkin, driving mitochondrial peripheral fission. (b) Cluster analysis of differentially expressed genes (DEGs) between the control group and the stress susceptibility group. (c) Differential protein interactions were analyzed in the stress susceptibility group compared with the control group. (d) Coq4 and Parkin expression on incubation with Glu (100 μM) for 1 h, and incubation with Glu (100 μM) and Mito-TEMPO (10 μM) for 1 h. (e) Altered mitochondrial Ca^2+^ homeostasis revealed by gene ontology (GO) enrichment detection. (f) Ca^2+^ concentration in control and stress susceptibility group mice detection by AVV-hSyn-GCamp6s. (g) Confocal imaging of O_2_^•–^ for mitochondria in primary neurons using probe RB-FM (10 μM, λ_ex_ = 561 nm, λ_em_ = 580–680 nm), and confocal imaging of morphological changes of mitochondria in primary neurons using MitoTracker Deep Red (100 nM, λ_ex_ = 644 nm, λ_em_ = 660–760 nm), control, incubation with Glu (100 μM) for 1 h, and incubation with Glu (100 μM) and EGTA (1 mM) for 1 h. Scale bar: 10 μm.

### Elevated Mitochondrial Ca^2+^ Concentrations Responsible for Elevated mtO_2_^•–^

Interestingly, we also found an elevated expression of the mitochondrial calcium uniporter 2 (Miu2) in hippocampal mitochondria of stress-susceptible mice (Figure 4b). Notably, the gene ontology (GO) enrichment analysis indicated positive alterations in mitochondrial Ca^2+^ homeostasis in these mice (Figure 4e), implying that Ca^2+^ concentrations may be upregulated. Subsequently, we injected AVV-hSyn-GCamp6s into the hippocampus and confirmed that Ca^2+^ concentrations in the hippocampus of stress-susceptible mice were indeed elevated (Figure 4f). Mitochondria serve as crucial intracellular Ca^2+^ receptors and storage organelles. When intracellular Ca^2+^ concentrations rise, Ca^2+^ flows into mitochondria through Miu2. Mitochondria are particularly sensitive to changes in Ca^2+^ levels, and elevated Ca^2+^ can promote ROS generation.^44–46^ Therefore, we speculate that the elevation of mtO_2_^•–^ within the hippocampus of stress-susceptible mice may be attributed to elevated Ca^2+^ concentrations in mitochondria. Next, to further verify the effect of elevated intracellular Ca^2+^ on the mitochondria, we cultured primary neurons derived from fetal rat hippocampus *in vitro*. Since Glu acts as an agonist of *N*-methyl-d-aspartate Glu receptors (NMDARs), it mediates the inward flow of Ca^2+^ by activating these channels in neurons. Consequently, we used Glu to stimulate an increase in intracellular Ca^2+^. As expected, Glu-induced elevations in neuronal Ca^2+^ concentrations (Figure S13) were followed by increases in mitochondrial Ca^2+^ concentrations (Figure S20). This suggests that elevated neuronal Ca^2+^ in the hippocampus of stress-susceptible mice also contributes to increased mitochondrial Ca^2+^ concentrations.

To further verify whether the Glu-induced elevation of Ca^2+^ was responsible for the elevated mtO_2_^•–^, we used ethylene glycol tetraacetic acid (EGTA, a Ca^2+^ chelator) to chelate Ca^2+^ from the medium prior to Glu stimulation. Imaging results indicated that Ca^2+^ removal effectively prevented the increase in mitochondrial O_2_^•–^ caused by Glu stimulation (Figure 4f). Moreover, Ca^2+^ removal also prevented the occurrence of mitochondrial peripheral fission caused by Glu incubation (Figure 4f). This suggests that Glu-mediated elevated Ca^2+^ can cause O_2_^•–^-dependent impairment of mitochondrial function, triggering the onset of mitochondrial peripheral fission. It also suggests that elevated hippocampal Ca^2+^ concentrations in stress-susceptible mice promote elevated mtO_2_^•–^, which mediates the rise in mitochondrial peripheral fission. This, in turn, contributes to a decrease in synaptic plasticity, highlighting its role in the development of depression (Figure 5).

**Figure 5:**
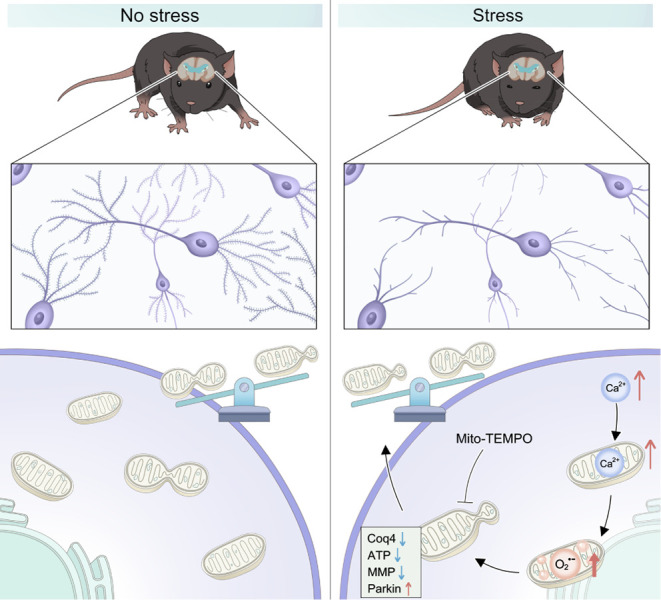
mtO_2_^•–^-dependent mitochondrial peripheral fission increase contributes to hippocampal synaptic dysfunction in stress-susceptible mice. Elevated Ca^2+^ levels in hippocampal neurons of stress-susceptible mice, which triggers mtO_2_^•–^-dependent reductions in Coq4, ATP, MMP, and elevations in Parkin, driving mitochondrial peripheral fission and reducing synaptic plasticity.

Reduced ATP production caused by mitochondrial dysfunction has been observed in preclinical animal models of depression.^10^ However, the specific features and molecular mechanisms of mitochondrial damage in hippocampal neurons remain obscure. Alterations in mitochondrial morphology and metabolism are closely associated with the dynamics of ROS flux.^38^ Therefore, clarifying the role of ROS, especially O_2_^•–^, in hippocampal mitochondrial damage in stress-susceptible mice may be crucial to resolving the aforementioned uncertainties. However, the migratory nature of existing tools does not accurately reflect the location where O_2_^•–^ is generated and, therefore, exerts its biological effect. Thus, we exploited a new fluorescence sensing and labeling strategy for accurately determining the spatiotemporal information on O_2_^•–^ during mitochondrial damage. Of note, we demonstrated that mtO_2_^•–^-dependent mitochondrial peripheral fission, a central hallmark of mitochondrial damage, contributes to hippocampal synaptic dysfunction in stress-susceptible mice.

Mitochondrial fission and fusion are mediated by the expression levels of proteins associated with mitochondrial dynamics.^38^ However, in the hippocampus of stress-susceptible mice, mitochondrial peripheral fission increased without any corresponding changes in the expression of Drp1, Mfn2, Opa1, Mfn1, and Fis1. This suggests a specific enhancement of mitochondrial peripheral fission, while the overall level of mitochondrial fission remains unaffected. This is evidenced by the statistical analysis of the mitochondrial peripheral and intermediate fissions using electron microscopic images.

Impaired mitochondrial biogenesis and oxidative stress can be key drivers of mitochondrial fission.^47^ This aligns with our observation of decreased levels of Coq4, Mtatp8, Coq4, Uqcc3, Mrps21, and Mrps33, alongside an increase in Parkin (Figure 4b,c). Furthermore, in primary neurons, we observed that O_2_^•–^-dependent mitochondrial damage is accompanied by elevated Parkin and reduced Coq4, MMP, and ATP. These results suggest that elevated mitochondrial O_2_^•–^ mediates the onset of mitochondrial peripheral fission by reducing mitochondrial biogenesis and disrupting redox homeostasis. However, proteomic and transcriptomic analyses did not reveal an increase in other autophagy-associated proteins; on the contrary, we found a reduction in the levels of autophagy-related protein 101 (Atg101) and autophagy-related protein 4d (Atg4d) (Figure 4b). This indicates that the removal of damaged mitochondrial fragments may not occur via autophagy.

Furthermore, elevated Glu levels in the brains of depressed individuals lead to overactivation of NMDARs, which facilitate the influx of Ca^2+^ into neurons.^2,48,49^ In this work, we observed that elevated intracellular Ca^2+^ (Figure 4e) triggers Ca^2+^ accumulation within the mitochondria, thereby contributing significantly to ROS production (Figure S20). This indicates that elevated Ca^2+^ concentrations in hippocampal neurons of stress-susceptible mice may be the beginning of mitochondrial damage.

## Conclusion

In conclusion, mtO_2_^•–^ may contribute to depression through mediating mitochondrial damage in hippocampal neurons, where establishing spatiotemporal correlations between its dynamic changes and mitochondrial structural and functional alterations proves essential for elucidating specific pathological mechanisms. To address this, we have, for the first time, developed a fluorescence sensing and labeling strategy to precisely capture the spatiotemporal information of O_2_^•–^. The developed fluorescence-based imaging probe (RB-FM) could rapidly react (within 5 s) with O_2_^•–^ with high sensitivity (detection limit: 0.75 nM), triggering covalent fluorescent labeling with nearby biological nucleophiles. This facilitates high-precision *in situ* imaging of O_2_^•–^ during mitochondrial damage, along with changes of synaptic plasticity in hippocampal neurons of stress-susceptible mice. Significantly, we have identified an intriguing form of mitochondrial damage in hippocampal neurons of stress-susceptible mice: a marked increase in mtO_2_^•–^-driven mitochondrial peripheral fission, while overall mitochondrial fission and mitophagy remain unchanged. We further uncovered a depression-related cascading pathway in which elevated Ca^2+^ levels in the hippocampus of stress-susceptible mice led to increased mtO_2_^•–^, aberrant expression of mitochondria-associated proteins, and ultimately mitochondrial peripheral fission, which contributes to impaired synaptic plasticity. This work provides a mechanistic framework for O_2_^•–^ control of mitochondrial peripheral fission and demonstrates how redox signaling interacts with synaptic plasticity in depression.

## Supplementary Material



## Notes

Additional experimental data, including experimental details, synthesis of RB-FM, photophysical properties, and fluorescence imaging (PDF)
