# Supplementary material for: Superoxide Anion-Dependent Mitochondrial Fission Contributes to Hippocampal Synaptic Dysfunction in Stress-Susceptible Mice
Source: JACS Au. 2025 Sep 15;5(10):4695–705. doi: 10.1021/jacsau.5c00493 (PMC12569654; doi:10.1021/jacsau.5c00493)
Supplement: Supplementary file 1 [file au5c00493_si_001.pdf]

Supporting Information for Publication

Superoxide anion-Dependent Mitochondrial Division Contributes to Hippocampal Synaptic Dysfunction in Stress-Susceptible Mice

Xiwei Li,[a] Xue Xue,[a] Simiao Zhang,[a] Tony D. James,[a,c,d]* Ping Li,[a,e]* Xin Wang,[a]* and Bo Tang[a,b]*

[a] College of Chemistry, Chemical Engineering and Materials Science, Key Laboratory of Molecular and Nano Probes, Ministry of Education, Collaborative Innovation Center of Functionalized Probes for Chemical Imaging in Universities of Shandong, Institutes of Biomedical Sciences, Shandong Normal University, Jinan 250014, P. R. China. [b] Laoshan Laboratory, 168 Wenhai Middle Rd, Aoshanwei Jimo, Qingdao 266237, Shandong. [c] Department of Chemistry, University of Bath, Bath BA2 7AY, United Kingdom. [d] School of Chemistry and Chemical Engineering, Henan Normal University, Xinxiang, 453007, P. R. China. [e] College of Chemistry and Chemical Engineering, Northwest Normal University, Lanzhou 730070, People's Republic China.

***Corresponding Authors:**

Email: t.d.james@bath.ac.uk, xinwang@sdnu.edu.cn, lip@sdnu.edu.cn, tangb@sdnu.edu.cn

Materials and Methods

Materials and instruments

2-(4-Diethylamino-2-hydroxybenzoyl)benzoicacid and 1,7-Naphthalenediol were purchased from Shanghai Macklin Biochemical Co., Ltd. Hexamethylenetetramine was obtained from Shandong aex chemical technology Co., Ltd. N,N-Dimethylformamide, NaBH(OAc3), (Dimethylamino)sulfur trifluoride (DAST) and N-Phenyl-bis(trifluoromethanesulfonimide) were obtained from Shanghai Aladdin Biochemical Technology Co., Ltd. Mitochondrial Membrane Potential Assay Kit with JC-1 was purchased from Beyotime.

Absorption spectra were recorded on a UV-Visible spectrophotometer (Evolution 220, Thermo Scientific). Fluorescence spectra were obtained with a Hitachi F-4700 fluorescence spectrophotometer. CCK-8 assay was performed using a Triturus microplate reader. Confocal imaging was performed on Leica Super-Resolution Microscopy (STED). The mass spectra were obtained using the Bruker Maxis ultra-high-resolution-TOF MS system. ^{1}H NMR spectra were obtained at 400 MHz using Bruker NMR spectrometers, and ^{13}C NMR spectra were recorded at 100 MHz.

Preparation of stock solution of probe RB-FM and ROS/RNS

When using probe RB-FM to detect $O_2^{\bullet -}$, the preparation protocol for the probe stock solution is as follows: dissolve 6 mg of probe RB-FM in 10 mL of DMSO to prepare a 1 mM probe stock solution.

When using probe RB-FM to detect other analytes, the preparation protocol for the probe stock solution is as follows: dissolve 6 mg of probe RB-FM in 10 mL of ethanol to prepare a 1 mM probe stock solution.

$O_2^{\bullet -}$: $O_2^{\bullet -}$ was prepared using crown ether and KO_2 in dry DMSO by an ultrasonic method. The concentration of $O_2^{\bullet -}$ was determined from the absorption at 250 nm (ε = 2682 $M^{-1}cm^{-1}$).

$ONOO^-$: 0.6 M $NaNO_2$, 0.6 M HC1 and 0.7 M H_2O_2 were added simultaneously to a 3 M NaOH solution at 0 ºC. The concentration of peroxynitrite was determined using extinction coefficient of 1670 $M^{-1}cm^{-1}$ at 302 nm in 0.1 M NaOH (aq.).

H_2O_2: H_2O_2 solutions were accessed by dilution of 30% hydrogen peroxide aqueous solution, the concentration was determined from the absorption at 240 nm (ε = 43.6 $M^{-1}cm^{-1}$).

TBHP (tert-butyl hydroperoxide): TBHP solutions were accessed by dilution of 70 % TBHP aqueous solution.

•OH (hydroxyl radical): •OH was generated by the Fenton reaction of $FeCl_2$ (1.0 mM) and H_2O_2 (200 μM) in deionised water.

NO (Nitric oxide): NO was obtained from a stock solution prepared by sodium nitroprusside.

$^1O_2$ (Singlet oxygen): $^1O_2$ was prepared by the NaClO-H_2O_2 system.

NaClO: NaClO solutions were accessed by dilution of high-purity sodium hypochlorite solution, the concentration was determined from the absorption at 292 nm (ε = 350 $M^{-1}cm^{-1}$).

Experimental procedure for fluorescence measurement and selectivity screen towards $O_2^{\bullet -}$

Various concentrations of $O_2^{\bullet -}$ (0-5 μM) were added to probe RB-FM in DMSO. After 1 min the mixture was diluted to 1 mL with PBS buffer (10 mM, pH 7.4). The final concentration of RB-FM in PBS buffer solution (with 0.5 % DMSO) was 5 μM. $\lambda_{ex}/_{em}$ = 561/615 nm.

Blank: Probe RB-FM (5 μM) was diluted to 1 mL with PBS buffer (10 mM, pH 7.4). $O_2^{\bullet -}$: $O_2^{\bullet -}$ (1 μM) was added to RB-FM in DMSO, and after 1 min the mixture was diluted to 1 mL with PBS buffer (10 mM, pH 7.4). The final concentration of RB-FM in PBS buffer solution (with 0.5% DMSO) was 5 μM. Other ROS/RNS, metal ions, GSH, Cys, and Hcy: The appropriate them was added to RB-FM in PBS buffer solution (with 0.5% ethanol), and incubated for 30 min. The final concentration of RB-FM in PBS buffer solution (with 0.5% ethanol) was 5 μM. $\lambda_{ex}/_{em}$ = 561/615 nm.

LOD calculation

The limit of detection (LOD) was calculated using the well-established method (LOD = 3σ/K), where K is slope of the calibration curve, σ represents is the standard deviation of the blank sample (11 times) of F_{615} for RB-FM without addition of $O_2^{\bullet -}$. (Note F_{615} refer to the fluorescence emission wavelength peaks at 615 nm).

Cell culture

PC12 cells were cultured in RPMI 1640 supplemented with 10 % fetal bovine serum, 1 % penicillin and 1 % streptomycin at 37 °C (w/v) in an MCO-15AC incubator (SANYO, Tokyo, Japan) in 5 % CO_2/95 % air. One day before imaging, the cells were detached and placed in glass-bottomed dishes.

Hippocampi were isolated at embryonic day 16-18, rinsed in ice-cold PBS 1X. After mechanical dissociation, neurons were treated with trypsin for 10 min at 37 °C. Then, DMEM/F-12 (1:1) (Shanghai BasalMedia Technologies Co., Ltd., L370KJ) and were applied in a one-to-one ratio for trypsin inactivation, the cells were centrifuged 5 min at 1000 × g. Neurons were plated at an initial density of 125,000 cells/cm^2 in 20 mm glass coverslips. All plates were coated overnight with poly-D-

lysine (Sigma–Aldrich, P0899). Neurons were cultured in Gibco Neurobasal Medium (Fisher Scientific, 21103049) supplemented with 2% B-27, 200 mM L-glutamine, 5 mg/ml penicillin, and 12.5 mg/ml streptomycin.

Cytotoxicity assays1

Cell Counting Kit-8 (CCK-8) assays were carried out to evaluate the toxicity of RB-FM. PC12 cells (10^6 cells mL^{-1}) were seeded into 96-well microtiter plates with total volumes of 200 μL $well^{-1}$. After 24 h of incubation, various concentrations of RB-FM (0 M, 1×10^{-6} M, 2×10^{-6} M, 3×10^{-6} M, 5×10^{-6} M, 1×10^{-5} M, 2×10^{-5} M, 3×10^{-5} M and 5×10^{-5} M) were added, and the PC12 cells were cultured for another 24 h. Afterwards, 10 μL of CCK-8 solution was added to each well. After 4 h of incubation, the absorbance at 450 nm was measured using a Bio-Tek multimode reader.

Mouse models with depression-like behaviour

Male C57BL/6J mice, aged 7-8 weeks and weighing 18-21 g were obtained from Jinan Pengyue Laboratory Animal Breeding Co., Ltd.. The mice were allowed to acclimate for 1 week before the experiments. Animals were kept in a controlled environment with a stable temperature (22 ± 2 °C), and had free access to food and water. All animal care and experimental protocols complied with the Animal Management Rules of the Ministry of Health of the People’s Republic of China and were approved by the Animal Care Committee of Shandong Normal University (AEECSDNU2024127). The use of animals for the experiments followed the Guide for Care and Use of Laboratory Animals as adopted and promulgated by the National Institutes of Health.

The mice with depression-like behaviour were established by chronic unpredictable mild stress (CUMS). The mice (n = 35) were subjected to a variety of stresses independently, including 45° cage tilting, food or water deprivation, a soiled cage, noise exposure, temperature variation, clip mouse tail, continuous overnight illumination, and inversion of the light/dark cycle. These stresses were applied randomly. After 28 days, mice were separated into stress susceptibility and stress resilience populations based on the sucrose preference test, forced swimming test, tail suspension test and open-field test. For all animal studies, the tester was blind to the group allocation.

Sucrose preference test

Sucrose preference test was conducted using a two-bottle choice procedure. Before the sucrose preference test (SPT), mice were habituated to drink a 1% sucrose solution for 24 h with two bottles. Then, the sucrose solution was replaced with water for an additional 24 h. At the start of the test, mice were given access to the two bottles, one filled with sucrose solution and the other with water. The position of the water and sucrose bottles (left or right) was switched every 30 min for 3 h. Then the mice were left undisturbed, and their overnight fluid consumption was measured the next morning. The volume of sucrose or water of every bottle was recorded. The sucrose preference was defined as the ratio of the volume of sucrose to the total volume of sucrose and water consumed.

Forced swimming test

Forced swimming test (FST) as a 2 day program were carried out following references. In the forced swimming test (FST), each mouse was placed in a cylindrical tank (24 cm height × 10 cm diameter) filled to 16 cm with water at a temperature of 24 ± 1 °C. The mice could swim freely. On the first day, the mice represented an escape-like behavior and found an immobility posture that they could maintain their head above water easily for conserving energy. After rested for 24 h, the mice were subjected to 6 min of swimming, but only the last four minutes were considered in the analysis.

Tail suspension test

In the tail suspension test (TST), each mouse was suspended by the tail using adhesive scotch tape from a hook connected to a strain gauge that detected all the movements of the mouse and transmitted them to a central unit, which calculated the total duration of immobility during a 6-min test. However, only the last four minutes were considered in the analysis.

Open-field test

The open-field test apparatus contained a rectangular chamber (50 × 50 × 40 cm) constructed from opaque white plastic and was employed to assess locomotor activity and anxiety levels. The floor of the chamber was divided into a central area (35 × 35 cm) and a surrounding border zone. The mice were gently placed in the central area and allowed to explore for 5 min. A video camera was above the center of the apparatus and the recording was conducted using TOPView Behavior Analyzing System software (Clever Sys. Inc.).

Golgi staining

Hito Golgi-Cox OptimStain Kit (USA, Hitobiltec Inc.) has been used for Golgi staining of mice brains. Mice were anaesthetized with 10% chloral hydrate (350 mg/kg, intraperitoneal) and then brain tissues were extracted on ice as quickly as possible. The brains were submerged under mixed impregnation solution (solution 1: solution 2 = 1:1) for 2 weeks at

room temperature in the dark. Then, the brains were transferred to solution 3 and stored at 4°C shielded from light for 3 days. Coronal sections (100 μm) were cut on a freezing microtome and mounted onto gelatinized slides. After drying in the dark, solutions 4 and 5 were used to rehydrate the slides, followed by dehydrating the slides in 50%, 75%, 95% and 100% ethanol. Finally, the sections were cleared in xylene (2 times, 4 min each) and covered with undiluted xylene based resinous mounting media. Intact neurons (2-3 per animal) were carefully chosen for analysis and not obscured due to the precipitation of stain. The slides could be viewed after drying by bright field microscopy. The number of dendritic spines in 50 μm and the number of branches were analyzed with ImageJ software.

Transmission electron microscope

The mice were anesthetized and the hippocampi was quickly stripped and placed in a pre-cooled fixative solution of 2.5% glutaraldehyde. Let sit for three minutes and then cut into 1 cubic millimeter pieces. Then, tissues were post-fixed with 2.5% glutaraldehyde for 2 h at room temperature. Subsequently, the samples were stored at 4 ℃. Subsequently, rinse 5-6 times using PBS. Fix with osmium acid for 1-1.5 hours under light-proof conditions. It was again rinsed 3-4 times with PBS. This was followed by gradient dehydration using different concentrations of ethanol. The samples were embedded after permeabilization using a mixture of acetone and resin. Next, the embedded samples were sectioned (70 nm) and stained. After drying, the samples were imaged using a transmission electron microscope (Hitachi HT-7800) operating at 80 KV.

Western blot assay

All the pre-treated cells were lysed in cell lysis buffer containing the protease inhibitor PMSF. The cell lysates were centrifuged at 12,000 rpm for 10 min at 4 °C. The hippocampus were sonicated in RIPA lysis buffer containing protease and phosphatase inhibitors. The samples were then centrifuged at 12,000 rpm for 20 min at 4 °C. Protein concentrations were measured using the BCA Protein Assay Reagent with BSA to establish a standard curve. Equal proteins were lysed in SDS sample buffer (62.5 mM Tris-HCl pH 6.8, 2 % SDS, 6 % glycerol, 0.005 % bromophenol blue, and 2.5 % 2-mercaptoethanol) and then boiled for 5 min at 95 °C. Proteins were separated using SDS-polyacrylamide gel electrophoresis and were then transferred to polyvinylidene difluoride membranes (Merck Millipore, Darmstadt, Germany) at 100 V for 1 h. Membranes were blocked with 5 % non-fat milk in Tris-buffered saline (TBS) containing 0.1 % Tween-20 (TBS-T) (20 mM Tris pH 7.5, 137 mM NaCl, and 0.1 % Tween 20) for 60 min and then incubated overnight at 4°C with the antibody. Then the membranes were washed with TBS-T, they were incubated with horseradish peroxidase (HRP)-conjugated anti-rabbit secondary antibodies (Abcam) at room temperature for 1 h. Then the samples were washed with TBS-T, protein bands were detected by using Immobilon Western Chemiluminescent HRP Substrate with the luminescent image analyzer (ChemiDoc MP, BIO RAD).

Mitochondrial membrane potential

To determine MMP, the JC-1 assay kit (Beyotime) was employed following the manufacturer′s protocol. Initially, the mitochondria of hippocampi were extracted using the mitochondria isolation kit for tissue (Med. Chem. Express.). Subsequently, the mitochondria were incubated with JC-1 for 30 min in a 5% CO_2 incubator at 37 °C to detect Δψm after washed with PBS. Fluorescence signals were measured at 490 nm excitation/530 nm emission for the monomer and 525 nm excitation/590 nm emission for the aggregate of JC-1. Carbonyl cyanide 3-chlorophenylhydrazone was added as a positive control.

Detection of ATP concentration

ATP concentration was determined using the ATP viability detection kit (Med. Chem. Express.) following the manufacturer’s instructions. The mitochondria of hippocampi were extracted using the mitochondria isolation kit for tissue (Med. Chem. Express.). The mitochondrial homogenate was then divided into 96-well plates and 100 μl luciferin-luciferase reaction buffer was added. The luminescent signal was measured using the BioTek Synergy 2 luminometer (BioTek). The ATP concentration was determined by constructing a standard curve and normalized to protein levels.

Stereotaxic injections

Adjust the fixation bars to the slits in front of the animal's ears and place the animal in the stereotaxic device. Using a scalpel, make an incision of approximately 1.5 cm between the ears, and clean the surface of the skull with a cotton swab. Align the syringe tip on the stereotaxic device with the three axes of the fontanel point. Write down the coordinates. The point is considered as the zero point. Lift the syringe on the vertical axis so that the plane movement does not scratch the skull, then move the syringe tip to the correct position. Hippocampal CA1 injections require the following coordinates (in millimeters) relative to bregma: -2 mm for the anterior/posterior axis, ±1.8 mm for the lateral/medial axis, and -1.5 mm for the dorsal/ventral axis. lower the tip of the syringe until it touches the skull and mark the spot with a marker pen, and with a fine drill bit, drill a shallow hole in the skull. Draw 0.5 ml of concentrated lentivirus solution (AVV-hSyn-GCamp6s). Place the syringe over the hole and slowly lower it vertically until it reaches the surface of the skull, continuing to slowly place the

syringe into the brain. The digital pump was set to 0.05 µL/min (0.5 µL over 10 min) and the injection was started. Once the injection is complete, wait an additional 10 minutes to allow the material to diffuse into the brain. Slowly remove the syringe and observe for reflux.

RNA sequencing and data analysis

Total RNA was extracted from hippocampi in control group, stress susceptibility group, stress resilience group mice, respectively. The transcriptome sequencing and analysis were conducted by OE Biotech Co., Ltd. (Shanghai). Total RNA was extracted using the TRIzol reagent (Invitrogen, CA, USA). RNA purity and quantification were evaluated using the NanoDrop 2000 spectrophotometer (Thermo Scientific). RNA integrity was assessed using the Agilent 2100 Bioanalyzer (Agilent Technologies, Santa Clara, CA, USA). Then the libraries were constructed using VAHTS Universal V6 RNA-seq Library Prep Kit. IluminaS8 NovaSeq 6000 platform was used for sequencing after qualifying database inspection. Differential expression genes (DEGs) analysis was performed using the DESeq2 5. Q value < 0.05 and |log2FC| > 1 was set as the threshold for significant DEGs.

Proteomics analysis

Total protein from mice hippocampi was extracted and digested with trypsin. Proteins were labeled with iTRAQ labeling reagents (ABSCIEX, 4,381,663) and then subjected to Liquid Chromatography and tandem MS analysis. Protein analysis and relative iTRAQ quantification services were conducted by OE Biotech Co., Ltd. (Shanghai). GO term and KEGG pathway enrichment analyses were used for biological categorization of the significantly differentially expressed proteins.

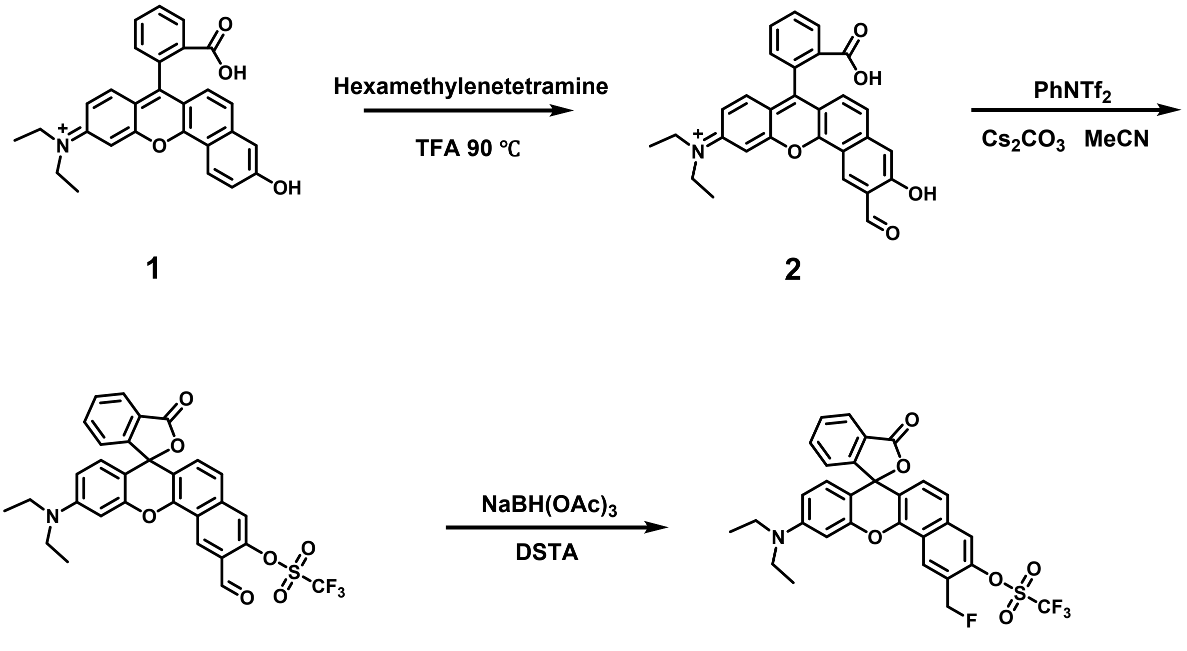

1

2

RB-SA

RB-FM

Figure S1 The synthetic route of RB-SA and RB-FM.

Synthesis of Compound 2

Compound 2 was prepared according to reported methods.[1] Under a nitrogen atmosphere, compound 1 (0.88 mg, 2.00 mmol) was added to trifluoroacetic acid (12 mL), followed by the addition of hexamethylenetetramine (0.28 g, 2.00 mmol). The mixture was stirred at 90 ℃ for 18 h. Then, Wahaha pure water (15 mL) was added and stirred at 90 ℃ for 15 min.

The solid crude product is obtained by filtration at the end of the reaction. The crude product was subsequently purified by column chromatography, eluting with dichloromethane/methanol (30:1, v/v) to afford compound 2 as a red solid (0.87 g, 1.46 mmol, 73%).

Synthesis of RB-SA

In an argon-flushed flask fitted with a septum cap, compound 2 (1.42 g, 3.53 mmol), Cs_2CO_3 (1.38 g, 4.24 mmol), and $PhNTf_2$ (1.89 g, 5.38 mmol) were dissolved in dry MeCN (40 mL). The reaction mixture was stirred for 2 h and then removal of solids by filtration. The solution was evaporated and the resultant crude product was purified by column chromatography eluting with dichloromethane/methanol (60:1, v/v) to afford compound 2 as a faint yellow solid (1.96 g, 3.28 mmol, 93%).

^{1}H NMR (400 MHz, $CDCl_3$): δ 10.74 (s, 1H), 9.04 (d, *J* = 12 Hz, 1H), 8.72 (d, *J* = 12 Hz, 1H), 8.07-8.09 (m, 1H), 7.62-7.69 (m, 3H), 7.14-7.16 (m, 1H), 7.05 (d, *J* = 8 Hz, 1H), 6.69 (d, *J* = 12 Hz, 2H), 6.49 (s, 1H), 3.39-3.44 (m, 4H), 1.22 (t, *J* = 8 Hz, 6H). ^{13}C NMR (100 MHz, $CDCl_3$): δ 187.13, 168.36, 152.05, 151.47, 151.03, 146.21, 134.11, 131.27, 130.50, 128.91. 128.38, 128.06, 125.80, 124.16, 122.92, 122.61, 121.46, 119.11, 118.73, 114.27, 11.39. ^{19}F NMR (400 MHz, $CDCl_3$) δ -72.65. HRMS (ESI): calcd for $C_{30}H_{22}F_3O_7NS$ $[M+H]^+$: 598.1142; found: 598.1122.

Synthesis of RB-FM

To a solution of RB-SA (60 mg, 0.01 mmol) in THF-MeOH (2 mL/2 mL) was added AcOH (6 mL, 0.01 mmol) and $NaBH(OAc)_3$ (26 mg, 0.12 mmol). The mixture was stirred for 1 h at ambient temperature under N_2 atmosphere, diluted with sat. NH_4Cl aq., and then extracted with EtOAc three times. The combined organic layer was dried over Na_2SO_4, and concentrated. The crude product was dissolved in CH_2Cl_2 (10 mL) and cooled to -20 °C. (Dimethylamino)sulfur trifluoride (DAST, 16 μL, 0.12 mmol) was added to the mixture in one portion and stirred at ambient temperature for 1 h. After addition of MeOH to quench excess of DAST, the mixture was evaporated. The crude product was purified by silica-gel column chromatography with CH_2Cl_2 eluent to yield 21 mg (35%) of 4 as faint yellow solid.

^{1}H NMR (400 MHz, DMSO-*d₆*): δ 8.93-8.96 (m, 1H), 8.07 (d, *J* = 8 Hz, 1H), 7.93-7.95 (m, 1H), 7.85 (d, *J* = 8 Hz, 1H), 7.74-7.81 (m, 2H), 7.31 (d, *J* = 8 Hz, 1H), 7.03 (d, *J* = 8 Hz, 1H), 6.80 (s, 1H), 6.55-6.62 (m, 2H), 5.97-6.03 (m, 1H), 5.85-5.91 (m, 1H), 3.39-3.44 (m, 4H), 1.14 (t, *J* = 8 Hz, 6H). ^{13}C NMR (100 MHz, DMSO-d_6): δ 169.53, 153.30, 152.18, 147.53, 145.97, 135.06, 134.24, 129.82, 129.09, 128.53, 126.83, 126.74, 125.71, 125.12, 124.01, 123.76, 120.29, 120.06, 119.66, 117.11, 114.40, 58.19, 55.96, 31.60, 22.66, 14.13, 12.40. ^{19}F NMR (400 MHz, DMSO-d_6) δ -73.13, -206.38. HRMS (ESI): calcd for $C_{30}H_{23}F_4O_6NS$ $[M+H]^+$: 602.1255; found: 602.1277.

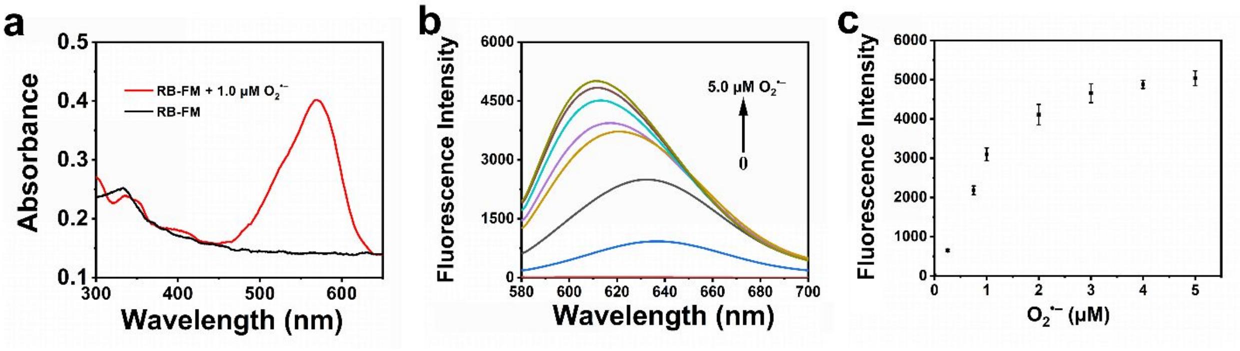

Figure S2 Spectral response of RB-FM toward $O_2^{\bullet-}$. (a) Absorption spectra and fluorescence spectra of RB-FM (5.0 μM) in PBS (pH 7.4) before and after incubation with $O_2^{\bullet-}$ (1.0 μM) within 1 min. (b) Fluorescence spectra of RB-FM (5.0 μM) after reaction with different concentrations of $O_2^{\bullet-}$ (0-5.0 μM). (c) The relationship between the fluorescence intensity of RB-FM (5.0 μM) at 615 nm and different concentrations of $O_2^{\bullet-}$ (0-5 μM). λ_{ex} = 561 nm.

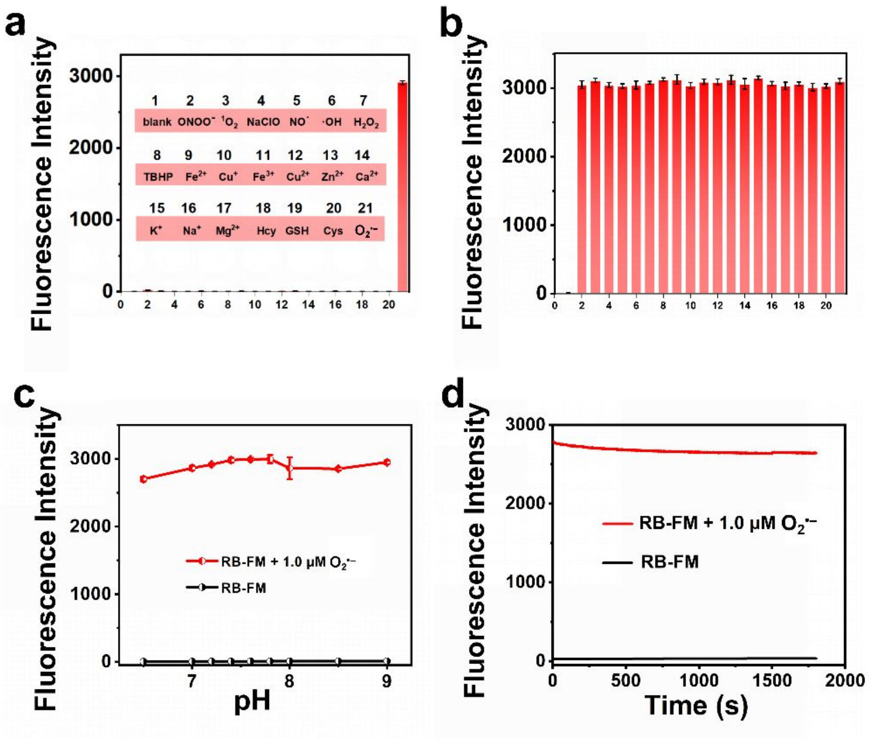

Figure S3 Spectral response of RB-FM. (a) Fluorescence response of RB-FM to ROS (50 μM $ONOO^-$, 50 μM $^1O_2$, 100 μM NaClO, 50 μM $NO^•$, 100 μM ·OH, 1 mM H_2O_2, 100 μM TBHP), metal ions (1 mM), GSH (5 mM), Cys (200 μM), Hcy (100 μM), and $O_2^{•-}$ (1 μM). (b) Fluorescence response of RB-FM to $O_2^{•-}$ in the present of other analytes. (c) Fluorescence intensity changes of RB-FM before and after reaction with $O_2^{•-}$ under various pH conditions. (d) Fluorescence intensity changes of RB-FM before and after its reaction with $O_2^{•-}$ over time (0-400 s). $\lambda_{ex}/_{em}$ = 561/615 nm.

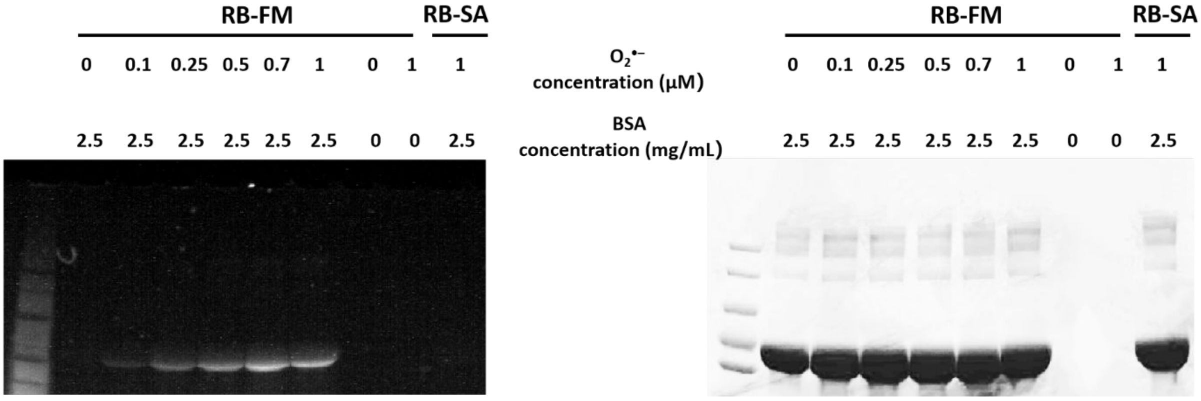

Figure S4 Evaluating the ability of probe RB-FM and RB-SA to anchor BSA after responding to $O_2^{\bullet-}$.

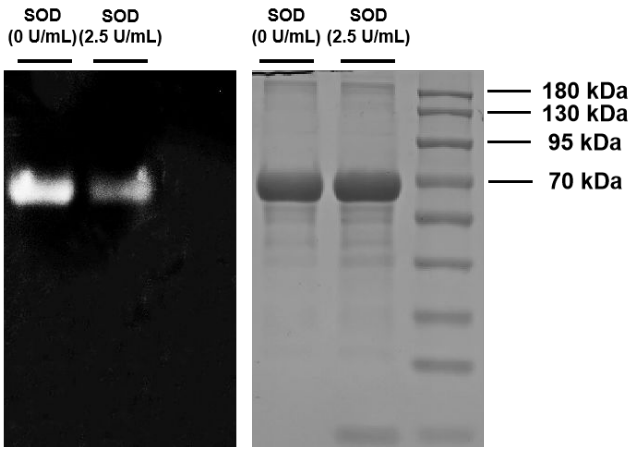

Figure S5 Evaluating the ability of probe RB-FM to anchor BSA (1 mg/mL) in response to $O_2^{\bullet -}$ (produced by xanthine oxidase, 5U/mL) without (the left band) or with (the right band) the addition of SOD.

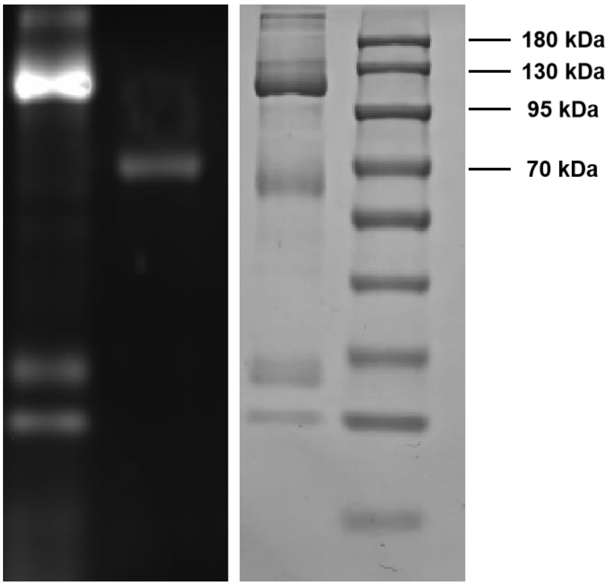

Figure S6 Evaluating the ability of probe RB-FM to anchor BSA (66 KDa, 0.025 mg/mL) or OX (160 KDa, 30 U/mL) in response to $O_2^{\bullet -}$.

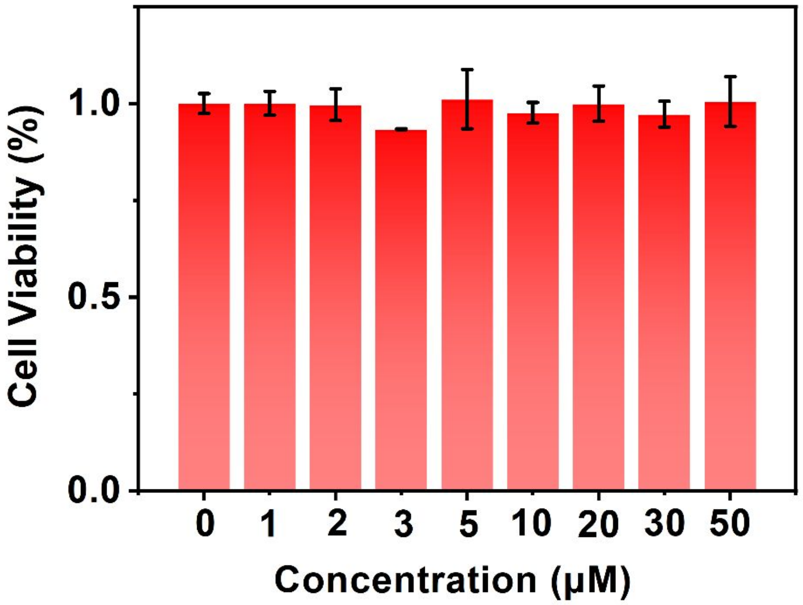

Figure S7 Effect of different concentrations of probe RB-FM after pretreated for 24 hours on cell viability was measured using CCK-8.

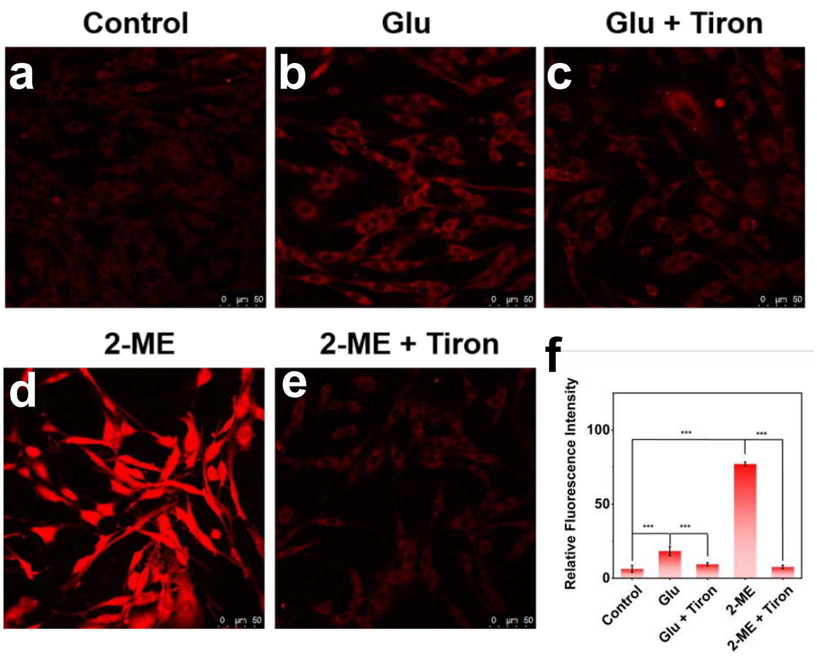

Figure S8 Confocal fluorescence imaging of $O_2^{\bullet-}$ levels in PC12 cells used probe RB-FM (10 μM). Fluorescence imaging of $O_2^{\bullet-}$ (λ_{ex} = 561 nm, λ_{em} = 580-680 nm) in control group (a), Glu (5 mM for 12h) group (b), Glu (5 mM for 12h) + Tiron (50 μM for 30 min) group (c), 2-ME (0.1 μg/mL for 15 min) group (d) and 2-ME (0.1 μg/mL for 15 min) + Tiron group (e). Relative fluorescence intensity output of each group (f). Note: The data are expressed as the mean ± SD. ***P < 0.001.

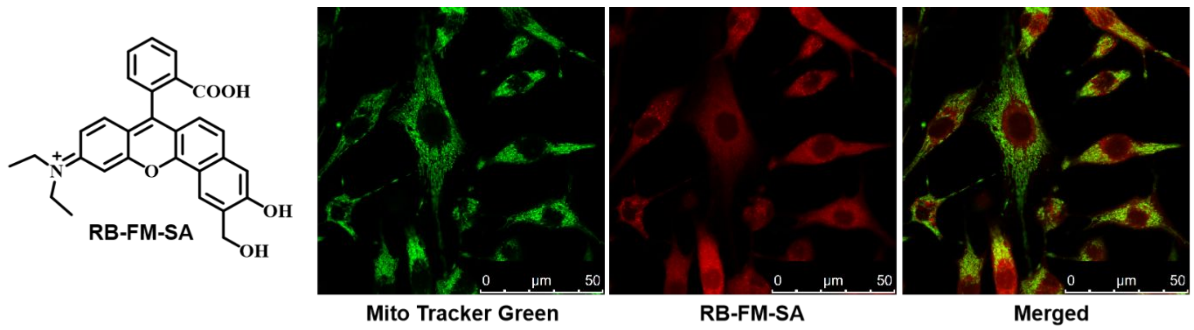

Figure S9 Evaluating the localization ability in mitochondria of probe RB-FM-SA (500 nM, λ_{ex} = 561 nm, λ_{em} = 580-680 nm) and MitoTracker green (100 nM, λ_{ex} = 488 nm, λ_{em} = 500-550 nm) in PC12 cells. Scale bar = 10 μm.

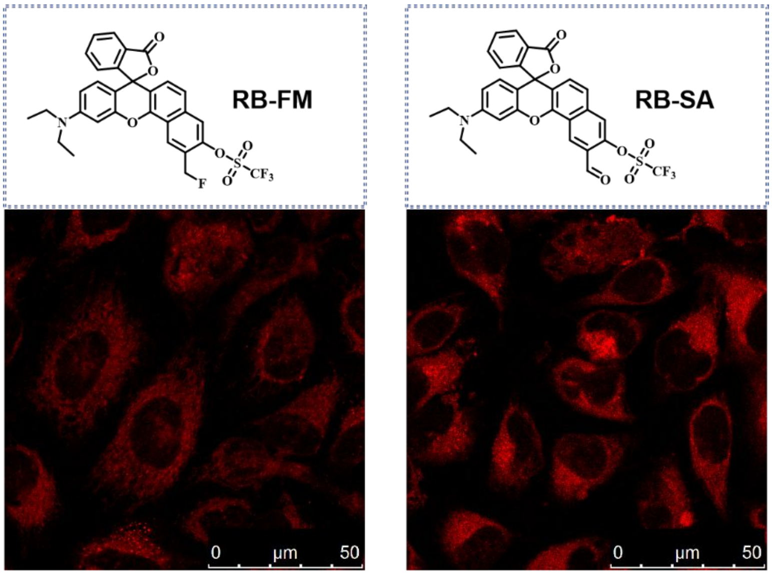

Figure S10 Cellular distribution patterns of probes RB-FM (10 μM, λ_{ex} = 561 nm, λ_{em} = 580-680 nm) and RB-SA (500 nM, λ_{ex} = 561 nm, λ_{em} = 580-680 nm). Scale bar = 10 μm.

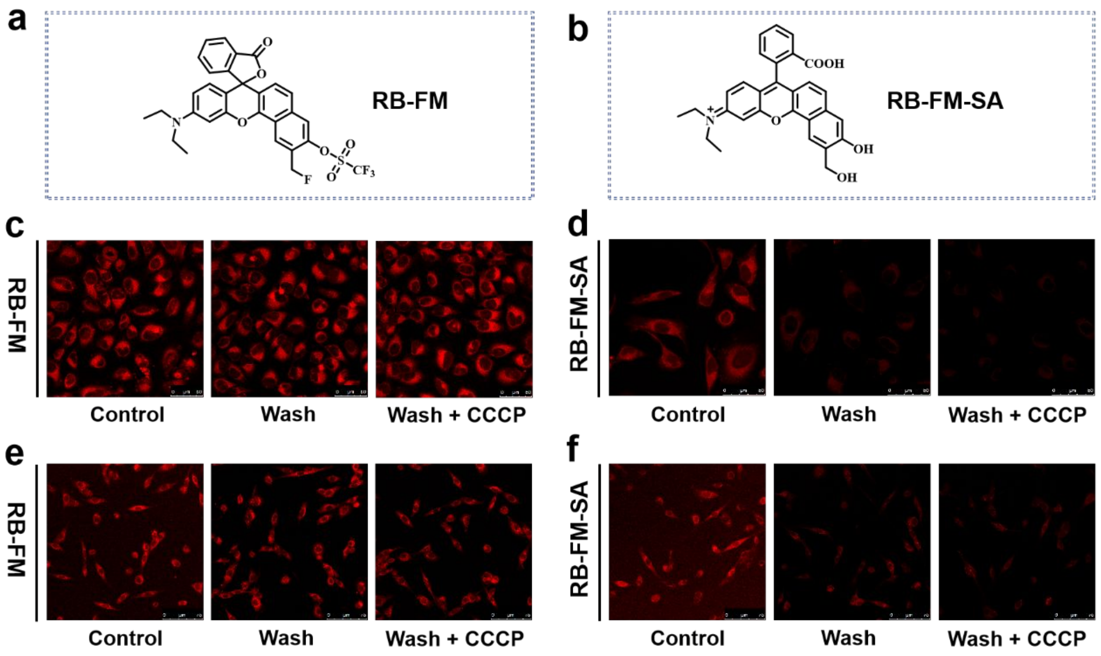

Figure S11 The structure of probe RB-FM, the activated probe (RB-FM-SA), and confocal fluorescence imaging. Spectral response of RB-FM toward $O_2^{\bullet-}$. (a) The structure of probe RB-FM. (b) The structure of RB-FM-SA. (c) Confocal fluorescence imaging of $O_2^{\bullet-}$ in HeLa cells was performed using probe RB-FM (10 μM, 60 min), with control, 10 washes, and subsequent incubation with CCCP (20 μM, 20 min) after an additional 10 washes. Scale bar = 10 μm. (d) Confocal fluorescence imaging of HeLa cells used probe RB-FM-SA (250 nM, 60 min), with control, 3 washes, and subsequent incubation with CCCP (20 μM, 20 min) after an additional 3 washes. Scale bar = 10 μm. (e) Confocal fluorescence imaging of $O_2^{\bullet-}$ in PC12 cells used probe RB-FM (10 μM, 60 min), with control, 10 washes, and subsequent incubation with CCCP (20 μM, 20 min) after an additional 3 washes. Scale bar = 15 μm. (f) Confocal fluorescence imaging of PC12 cells used probe RB-FM-SA (250 nM, 60 min), with control, 10 washes, and subsequent incubation with CCCP (20 μM, 20 min) after an additional 10 washes. Scale bar = 15 μm. λ_{ex} = 561 nm, λ_{em} = 580-680 nm.

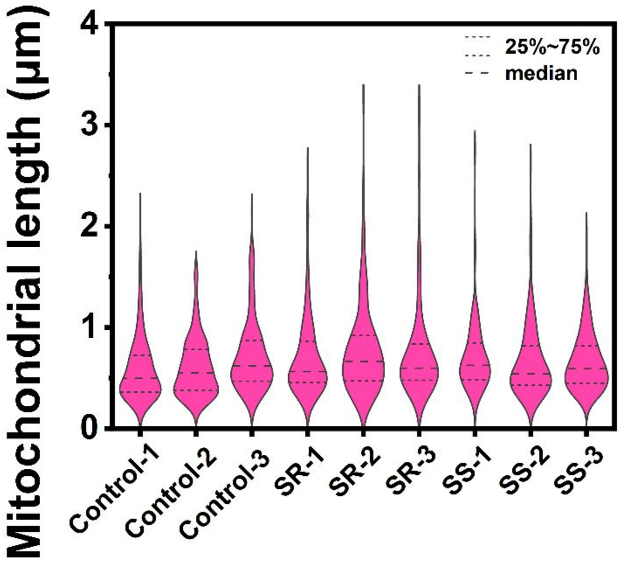

Figure S12 The size distribution of mitochondria in hippocampus of each group.

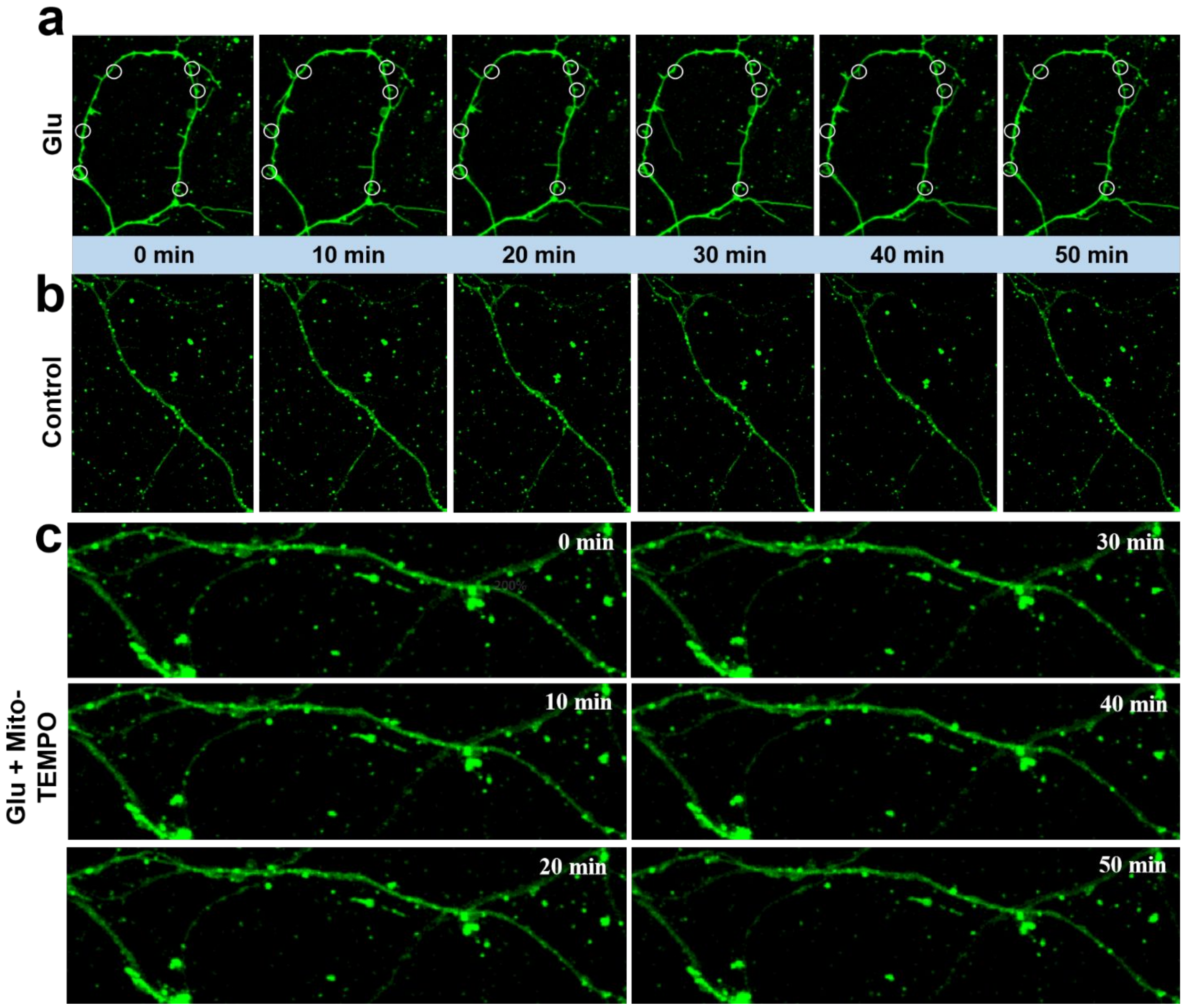

Figure S13 Confocal fluorescence imaging of changes in density and morphology of neuronal dendritic spines with dio. Use of dio to incubate neurons 30 min, and then add Glu (100 μM) (a), control (b), add Glu (100 μM) + Mito-TEMPO (50 μM) (c). λ_{ex} = 488 nm, λ_{em} = 500-550 nm.

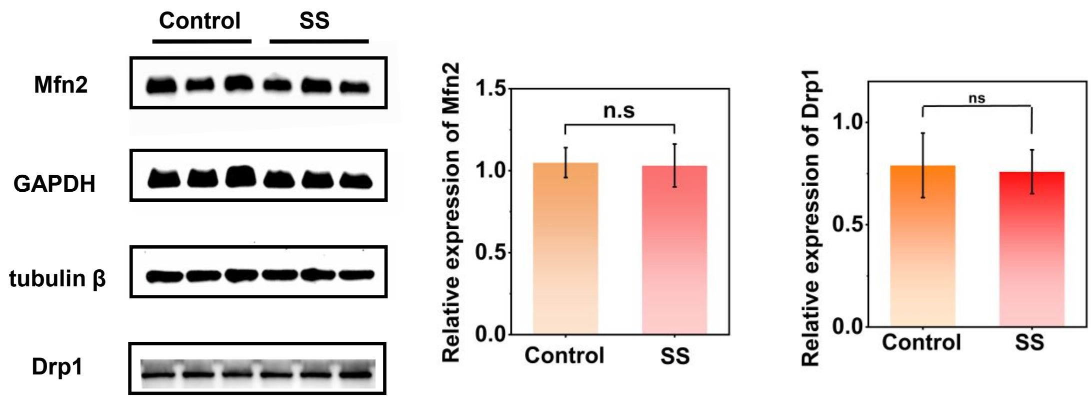

Figure S14 Expression levels of Mfn2 and Drp1 in the hippocampus of control and stress susceptibility mice.

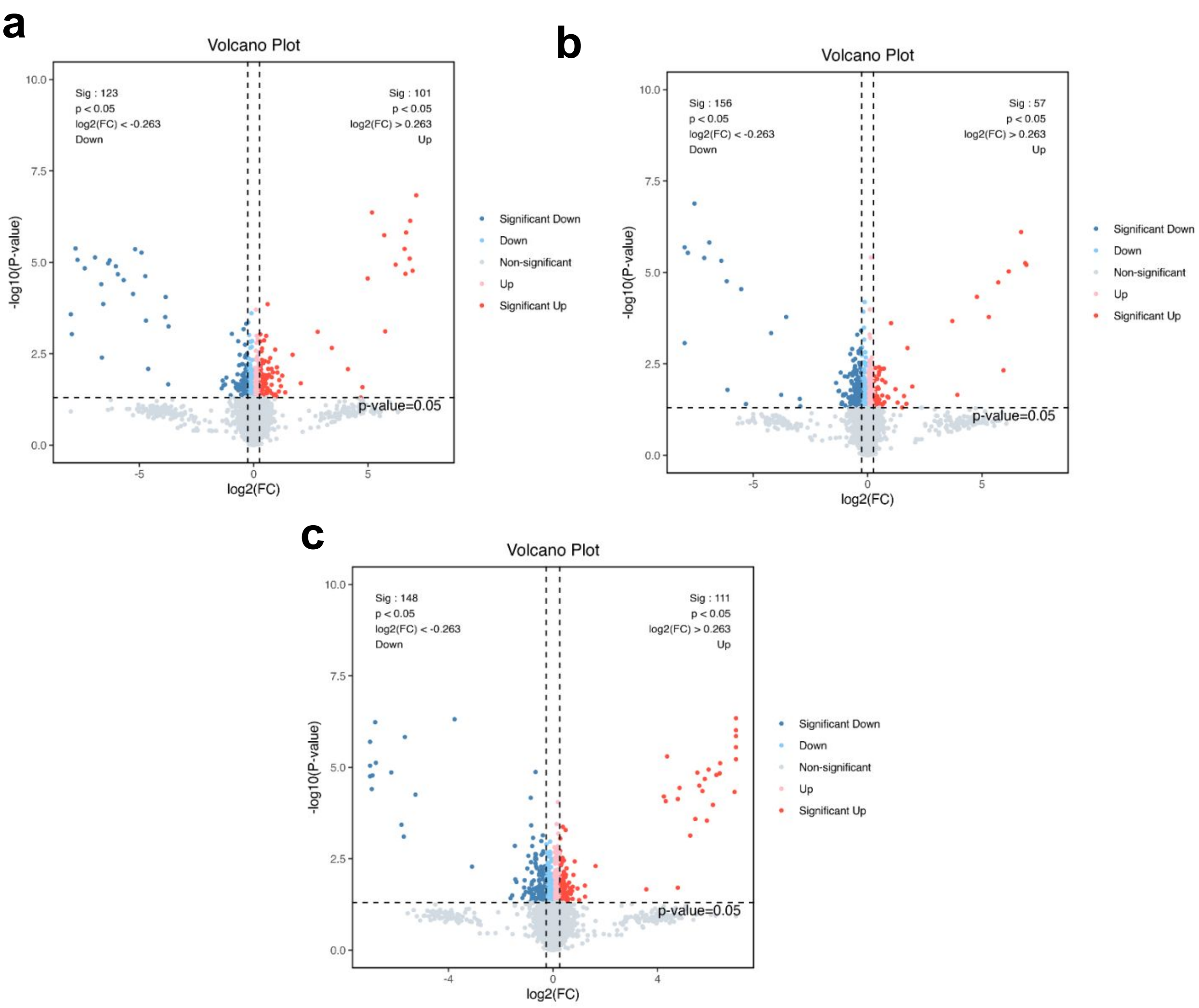

Figure S15 Differences in hippocampal protein expression in control and stress susceptibility mice. Volcano plots showing results of the control and stress resilience stress group (a), control and susceptibility group (b) and the resilient to stress and susceptibility group (c). DEGs were identified using q value < 0.05 and |log2FC| > 1 as the threshold parameters. Down-regulated DEGs are presented as blue dots while up-regulated ones are shown as red dots.

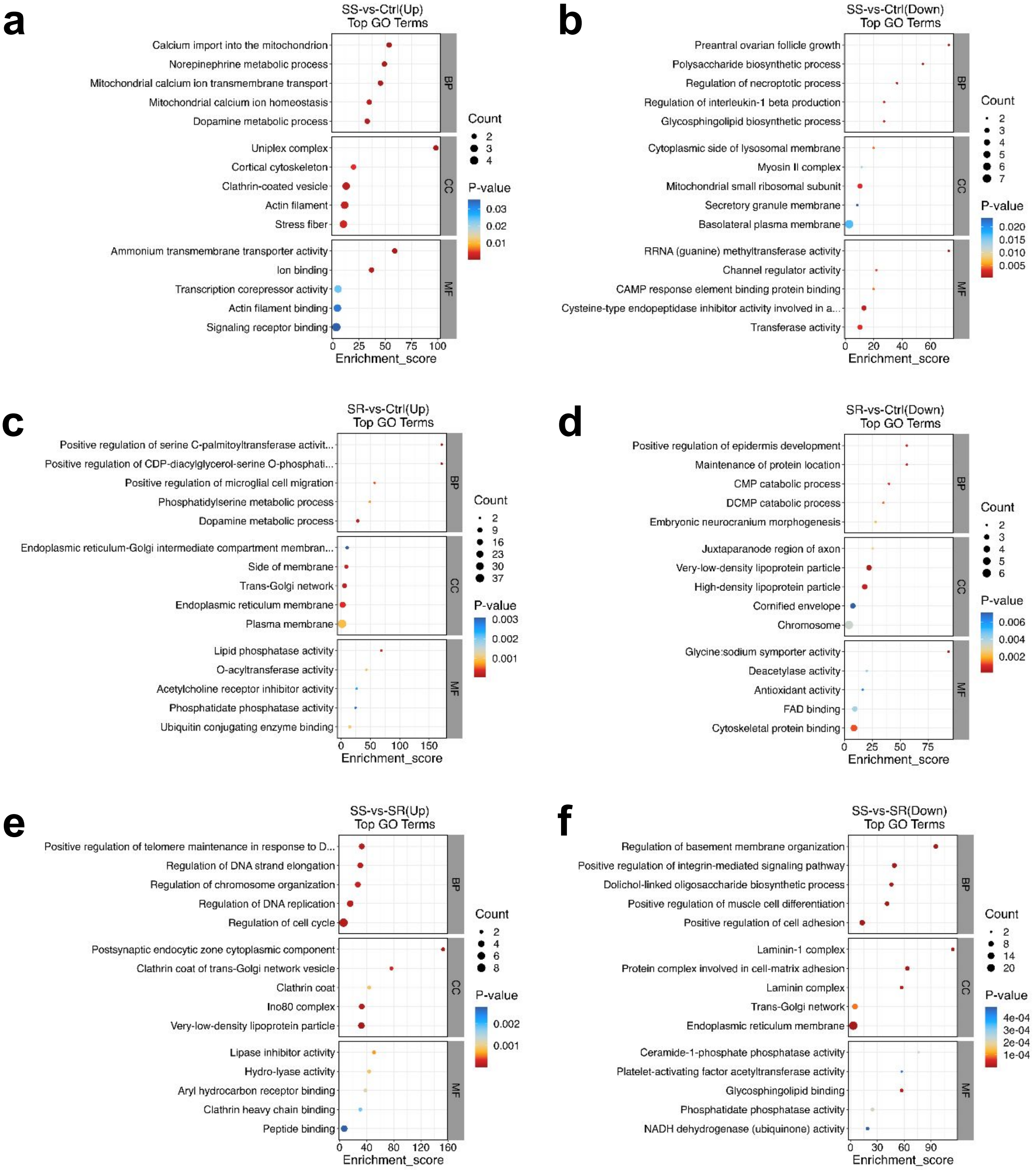

Figure S16 Go pathways top 15 of down-regulated and up-regulated proteins of the stress susceptible group compared and control group (a,b), resilience group compared control group (c,d), and stress susceptible group compared resilience to stress group (e,f). The horizontal coordinate Enrichment Score in the graph is the enrichment score, and the vertical coordinate is the information on the respective top 5 terms of BP/CC/MF. The larger the bubble, the more proteins are included in the entry, and the color of the bubble changes from blue to red, the redder the color is the smaller its enrichment p-value value is and the greater the degree of significance is.

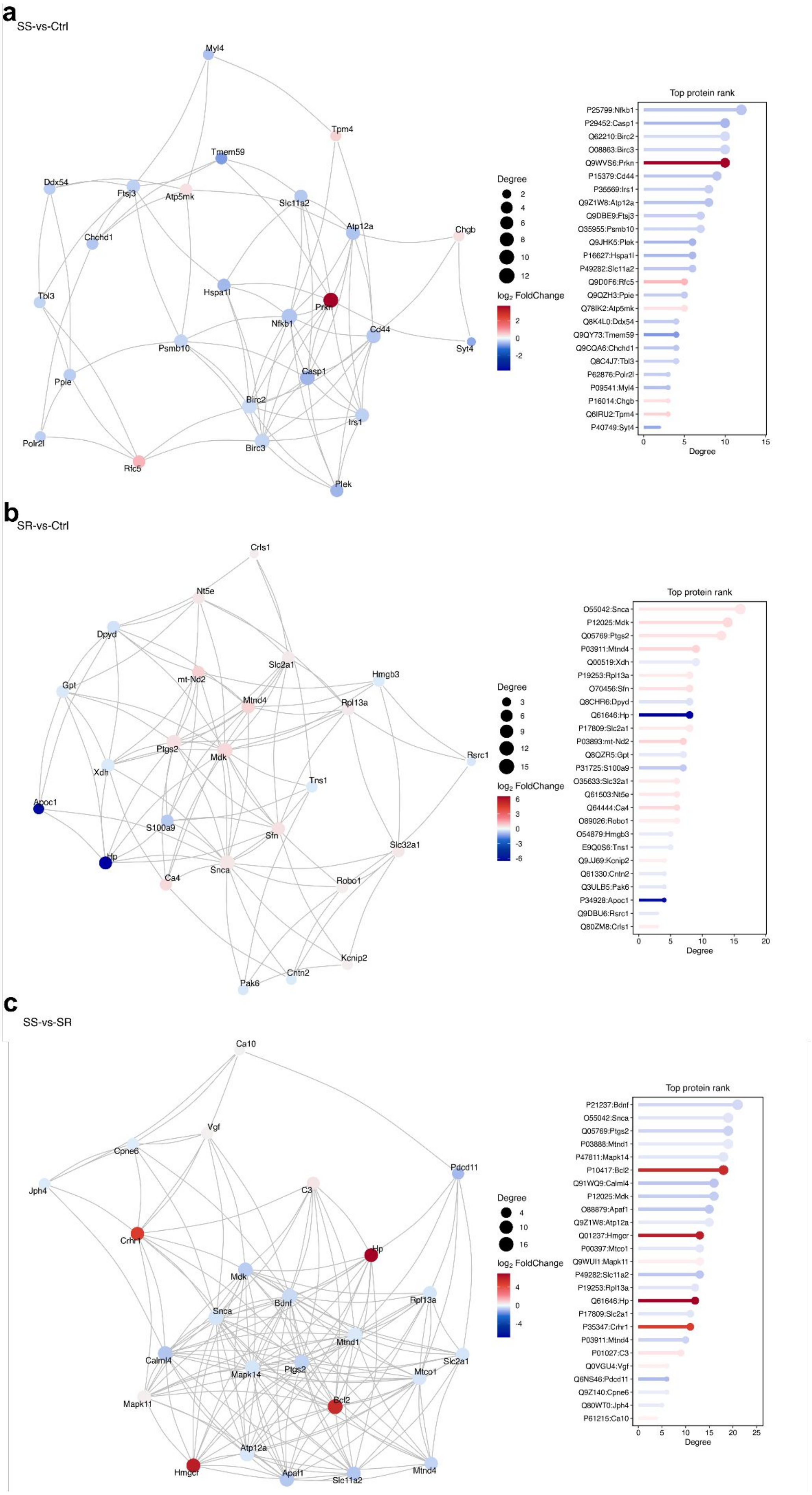
a
SS-vs-Ctrl
Myl4
Tpm4
Tmem59
Ddx54
Ftsj3
Atp5mk
Slc11a2
Atp12a
Chgb
Chchd1
Tbl3
Hspa1l
Prkn
Nfkb1
Cd44
Syt4
Psmb10
Casp1
Ppie
Birc2
Irs1
Polr2l
Birc3
Rfc5
Plek
Degree
2
4
6
8
10
12
log2 FoldChange
2
0
-2
Top protein rank
P25799:Nfkb1
P29452:Casp1
Q62210:Birc2
O08863:Birc3
Q9WVS6:Prkn
P15379:Cd44
P35569:Irs1
Q9Z1W8:Atp12a
Q9DBE9:Ftsj3
O35955:Psmb10
Q9JHK5:Plek
P16627:Hspa1l
P49282:Slc11a2
Q9D0F6:Rfc5
Q9QZH3:Ppie
Q78IK2:Atp5mk
Q8K4L0:Ddx54
Q9QY73:Tmem59
Q9CQA6:Chchd1
Q8C4J7:Tbl3
P62876:Polr2l
P09541:Myl4
P16014:Chgb
Q6IRU2:Tpm4
P40749:Syt4
0
5
10
15
Degree
b
SR-vs-Ctrl
Crls1
Nt5e
Dpyd
Slc2a1
Hmgb3
mt-Nd2
Gpt
Mtnd4
Rpl13a
Ptgs2
Mdk
Rsrc1
Xdh
Tns1
Apoc1
S100a9
Sfn
Slc32a1
Hp
Ca4
Snca
Robo1
Kcnip2
Cntn2
Pak6
Degree
3
6
9
12
15
log2 FoldChange
6
3
0
-3
-6
Top protein rank
O55042:Snca
P12025:Mdk
Q05769:Ptgs2
P03911:Mtnd4
Q00519:Xdh
P19253:Rpl13a
O70456:Sfn
Q8CHR6:Dpyd
Q61646:Hp
P17809:Slc2a1
P03893:mt-Nd2
Q8QZR5:Gpt
P31725:S100a9
O35633:Slc32a1
Q61503:Nt5e
Q64444:Ca4
O89026:Robo1
O54879:Hmgb3
E9Q0S6:Tns1
Q9JJ69:Kcnip2
Q61330:Cntn2
Q3ULB5:Pak6
P34928:Apoc1
Q9DBU6:Rsrc1
Q80ZM8:Crls1
0
5
10
15
20
Degree
c
SS-vs-SR
Ca10
Vgf
Cpne6
Pdcd11
Jph4
C3
Crhr1
Hp
Mdk
Bdnf
Rpl13a
Snca
Mtnd1
Calml4
Slc2a1
Mapk14
Ptgs2
Mtco1
Mapk11
Bcl2
Atp12a
Hmgcr
Apaf1
Slc11a2
Mtnd4
Degree
4
10
16
log2 FoldChange
4
0
-4
Top protein rank
P21237:Bdnf
O55042:Snca
Q05769:Ptgs2
P03888:Mtnd1
P47811:Mapk14
P10417:Bcl2
Q91WQ9:Calml4
P12025:Mdk
O88879:Apaf1
Q9Z1W8:Atp12a
Q01237:Hmgcr
P00397:Mtco1
Q9WUI1:Mapk11
P49282:Slc11a2
P19253:Rpl13a
Q61646:Hp
P17809:Slc2a1
P35347:Crhr1
P03911:Mtnd4
P01027:C3
Q0VGU4:Vgf
Q6NS46:Pdcd11
Q9Z140:Cpne6
Q80WT0:Jph4
P61215:Ca10
0
5
10
15
20
25
Degree

Figure S17 Differential protein interactions were analyzed in the stress resilient and stress susceptible group compared with the control group. Susceptible to stress group compared and control group (a), resilience group compared control group (b), and stress susceptible group compared resilient to stress group (c). On the left side is the top 25 connectivity protein interactions network diagram, circles indicate differential proteins, red represents up-regulation, blue represents down-regulation, and the size of the circle represents high or low connectivity; on the right side is the top 25 connectivity protein expression histogram. The size of the circle represents the degree of connectivity, the larger the circle, the higher the degree of connectivity; on the right side, the histogram of Top25 connectivity protein expression is shown.

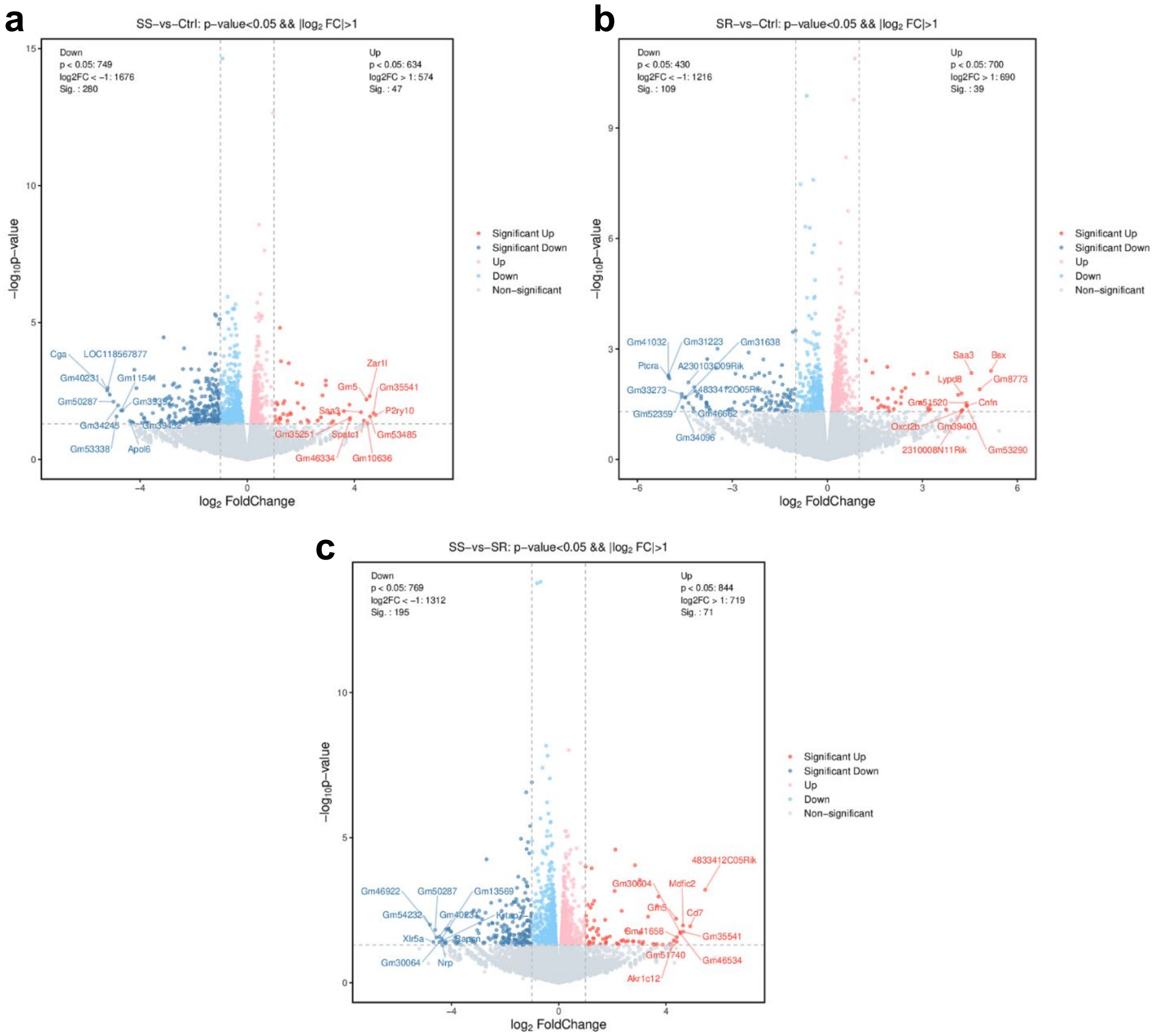

Figure S18 Differences in hippocampal transcript level in control and stress susceptible mice. Volcano plots showing results of the control and stress resilient group (a), control and susceptible group (b) and the resilient to stress and susceptible group (c). DEGs were identified using q value < 0.05 and $|log_2FC|$ > 1 as the threshold parameters. Down-regulated DEGs are presented as blue dots while up-regulated ones are shown as red dots.

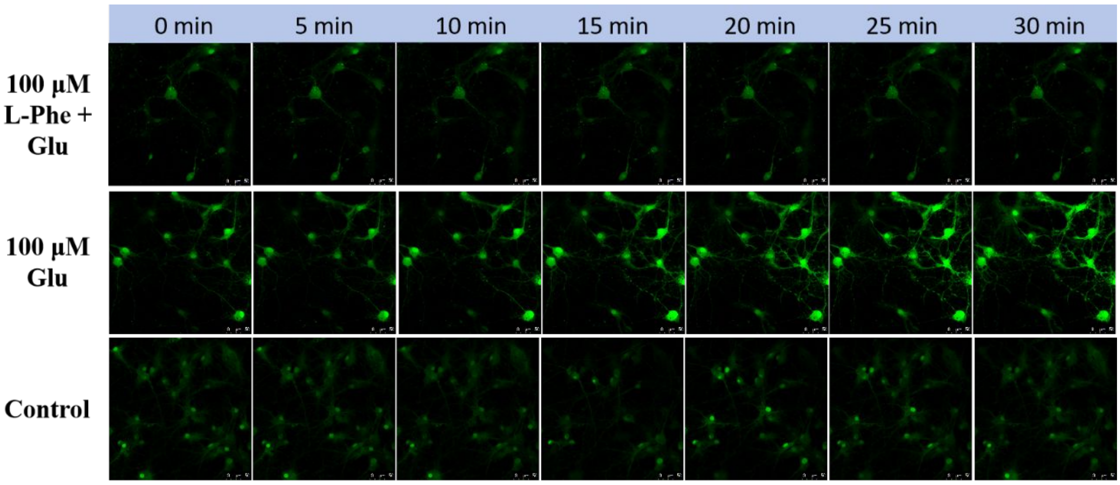

Figure S19 Confocal fluorescence imaging of Ca^{2+} levels using Fluo-3AM probe with primary neurons under the treatment of Glu or L-Phe and Glu. λ_{ex} = 488 nm, λ_{em} = 500-550 nm. Cells were incubated with Fluo-3AM for 15 min after removal of the medium, and then different stimuli were added, followed by fast-tracking of the fluorescence intensity of each group of cells for 30 min.

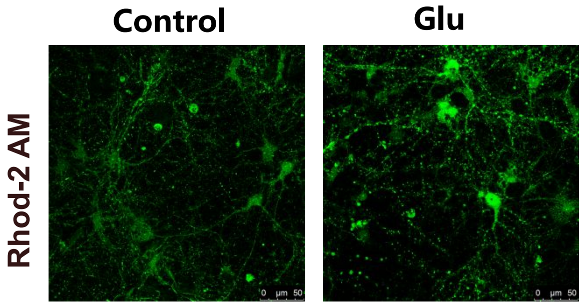

Figure S20 Confocal fluorescence imaging of Ca^{2+} levels using Fluo-2AM probe with mitochondria under the treatment of Glu. $λ_{ex}$ = 488 nm, $λ_{em}$ = 500-550 nm. Cells were incubated with a Fluo-2AM for 15 min after removal of the medium, and then different stimuli were added, followed by fast-tracking of the fluorescence intensity of each group of cells within 30 min.

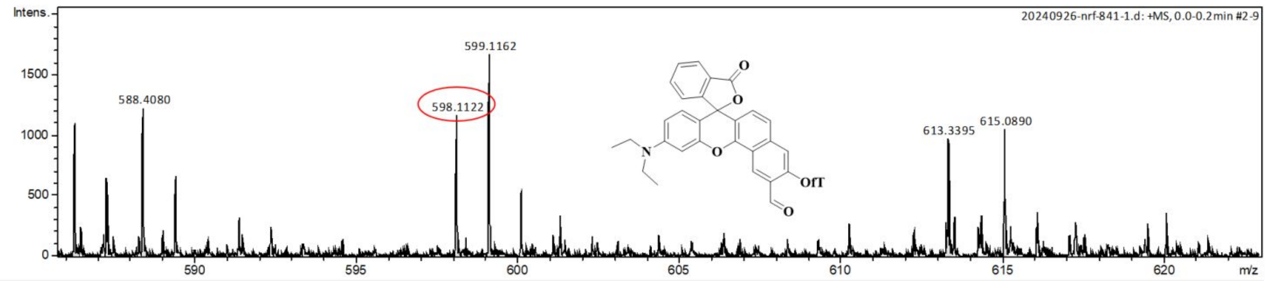

Figure S21 HRMS of RB-SA.

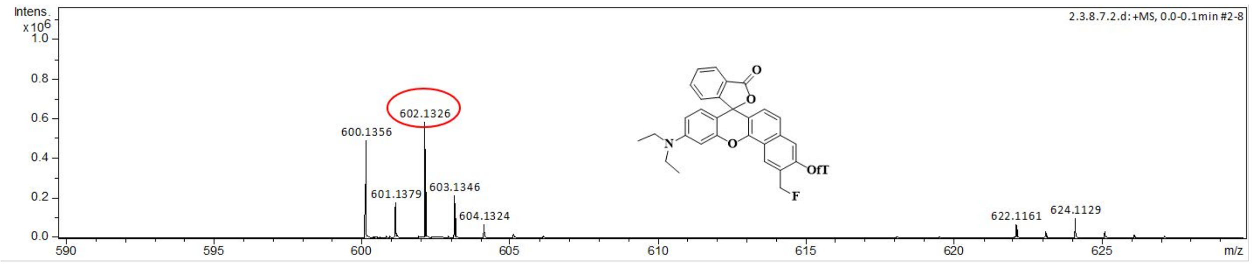

Figure S22 HRMS of RB-FM.

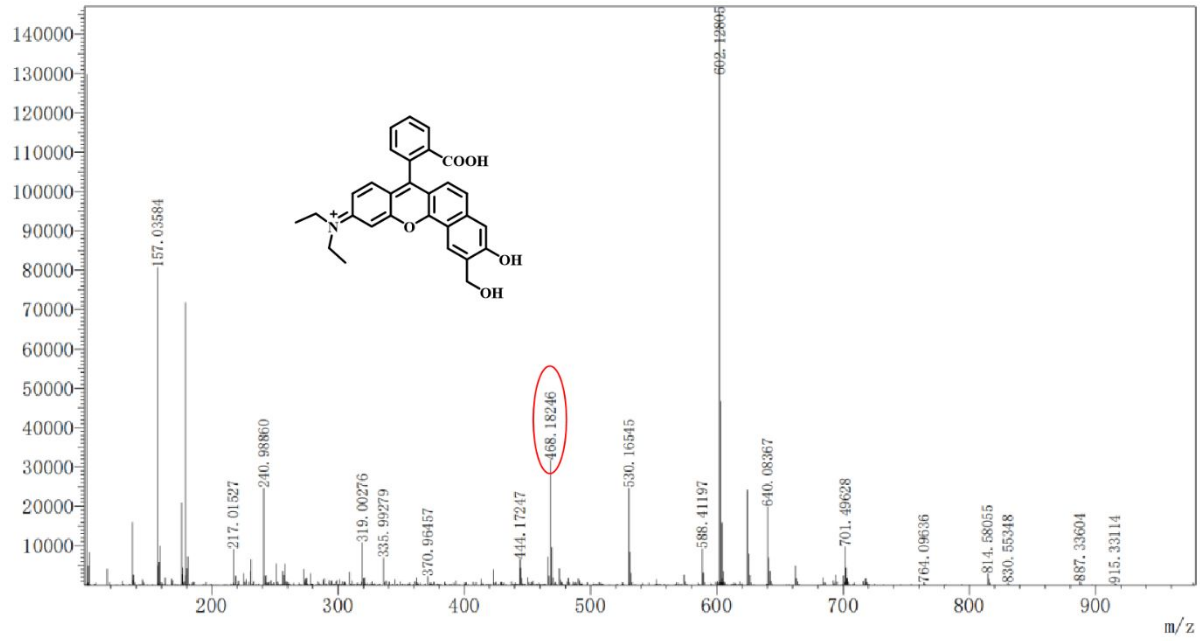

Figure S23 HRMS of RB-FM-SA.

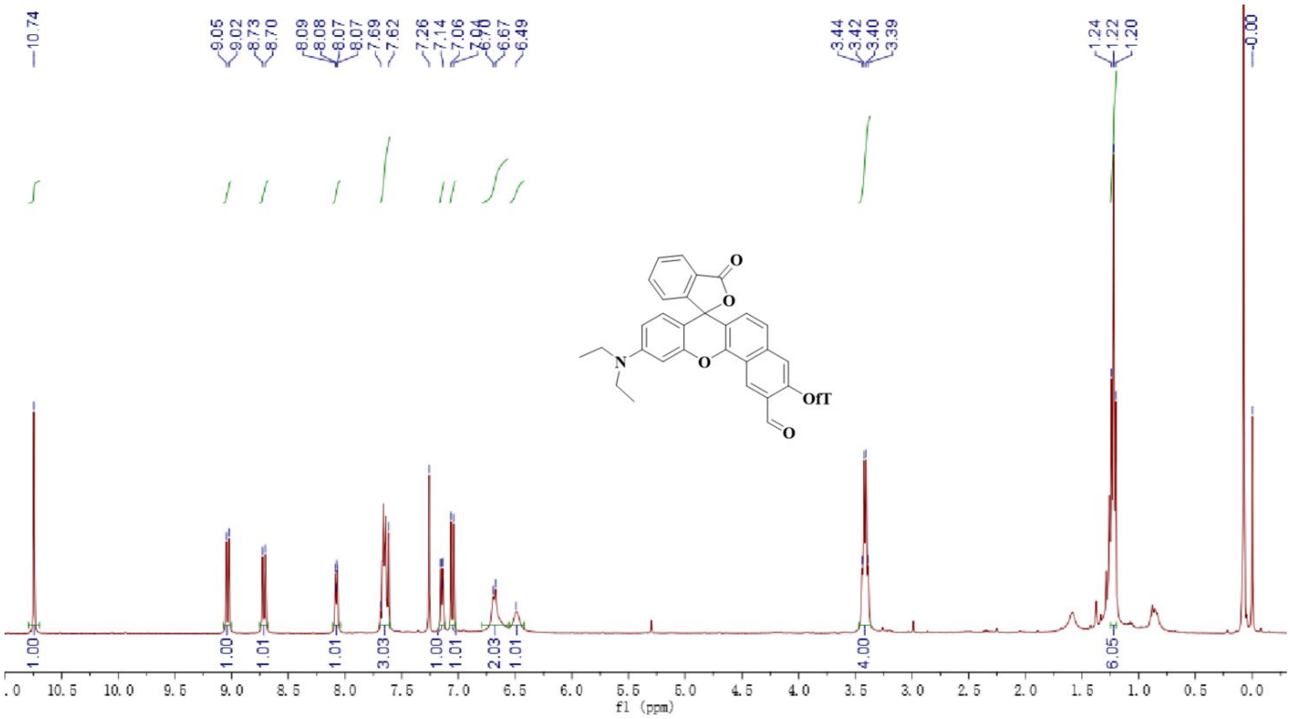

Figure S24 ^{1}H NMR (400 MHz, $CDCl_3$) of RB-SA.

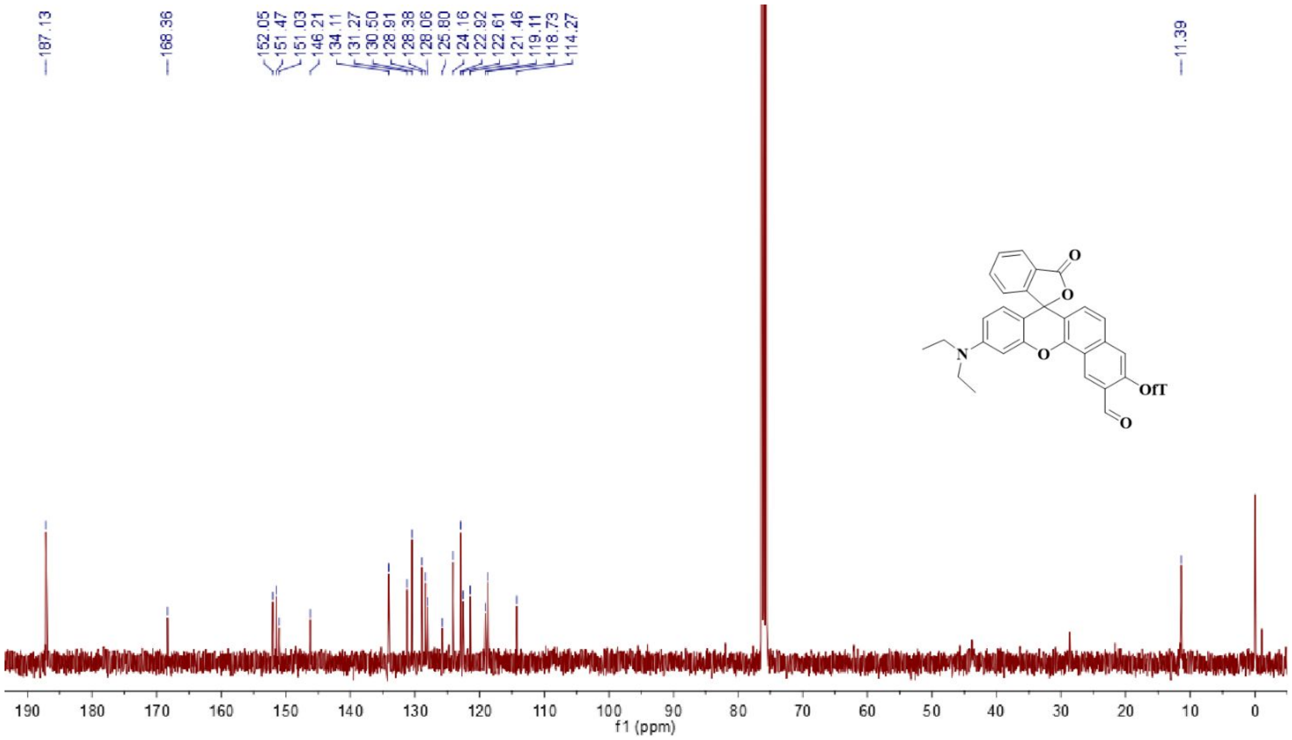

Figure S25 ^{13}C NMR (100 MHz, $CDCl_3$) of RB-SA.

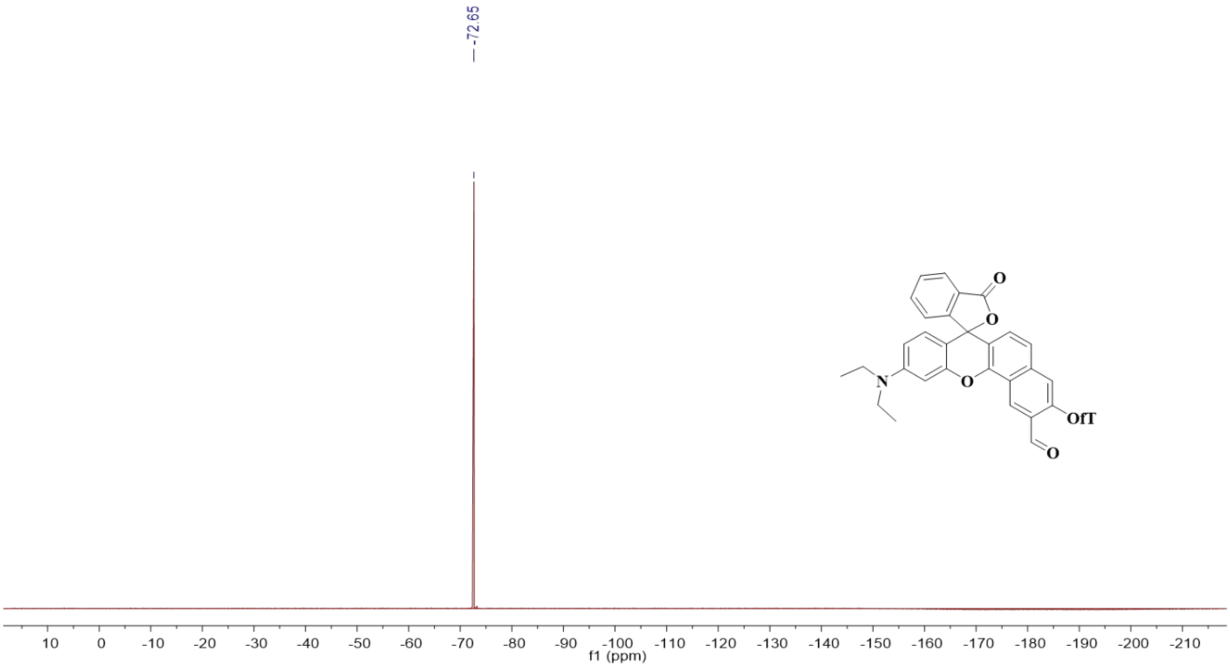

Figure S26 ^{19}F NMR (400 MHz, $CDCl_3$) of RB-SA.

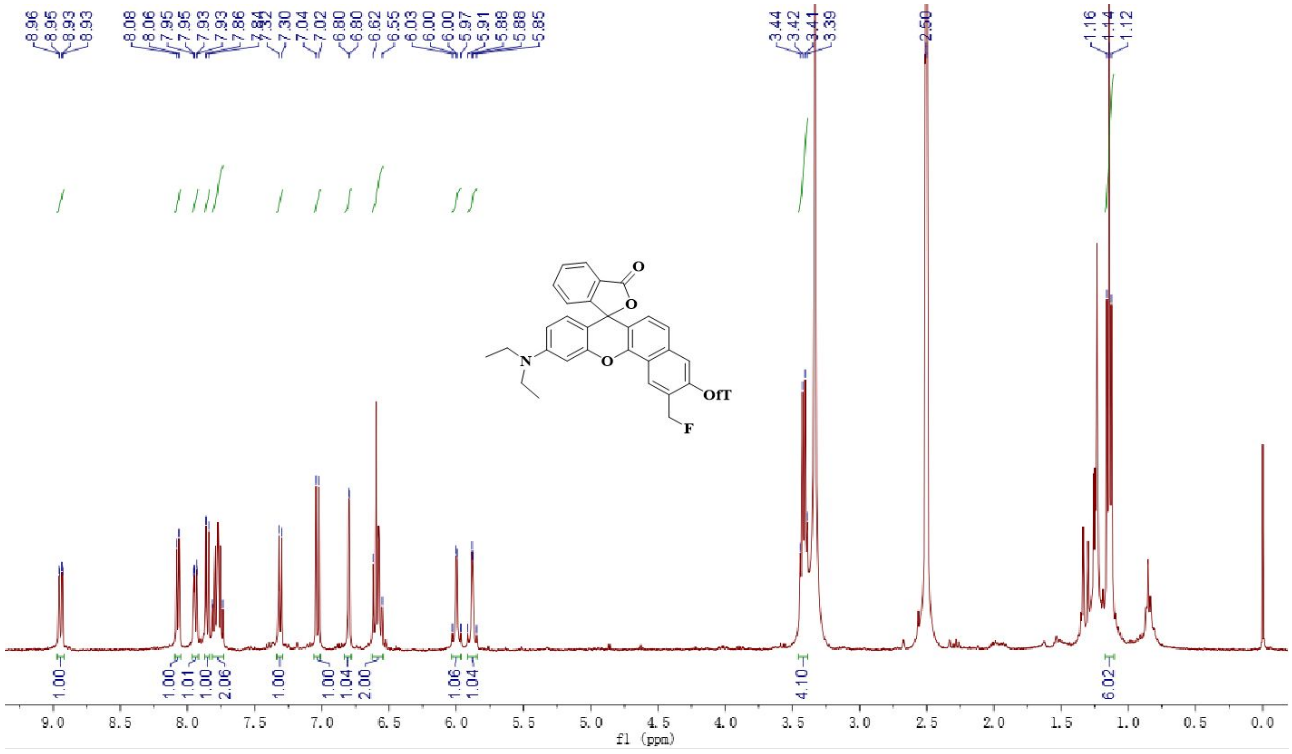

Figure S27 ^{1}H NMR (400 MHz, DMSO-d_6) of RB-FM.

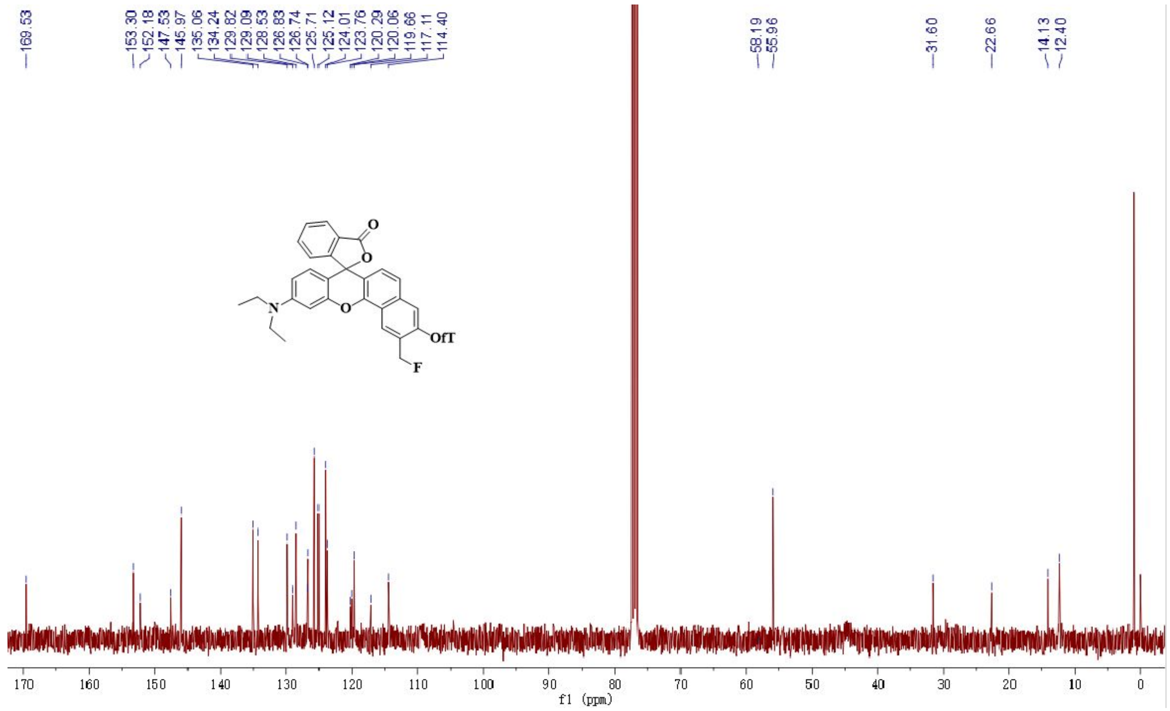

Figure S28 ^{13}C NMR (100 MHz, DMSO-d_6) of RB-FM.

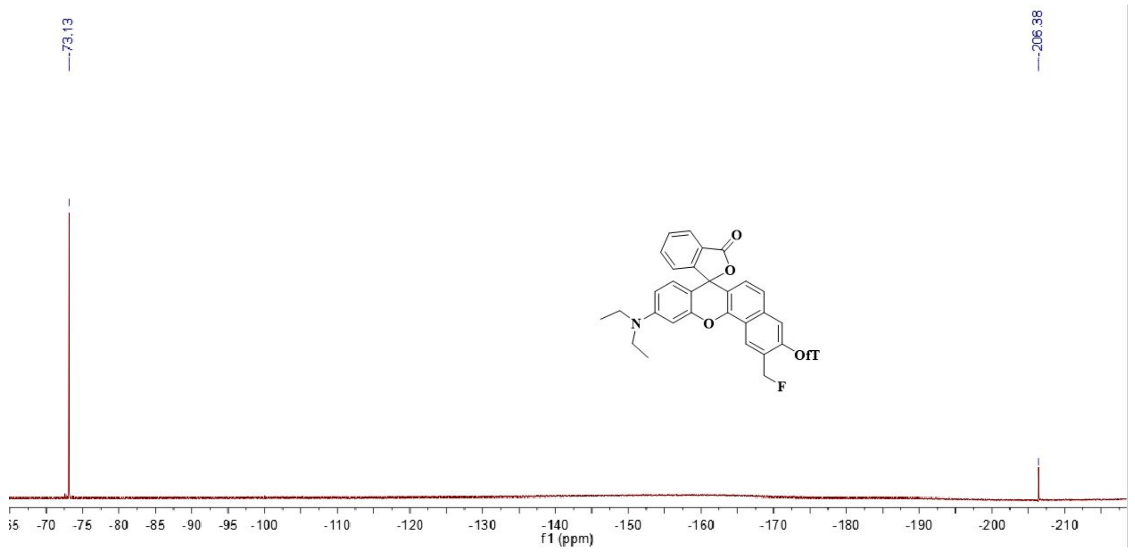

Figure S29 ^{19}F NMR (400 MHz, DMSO-d_6) of RB-FM.

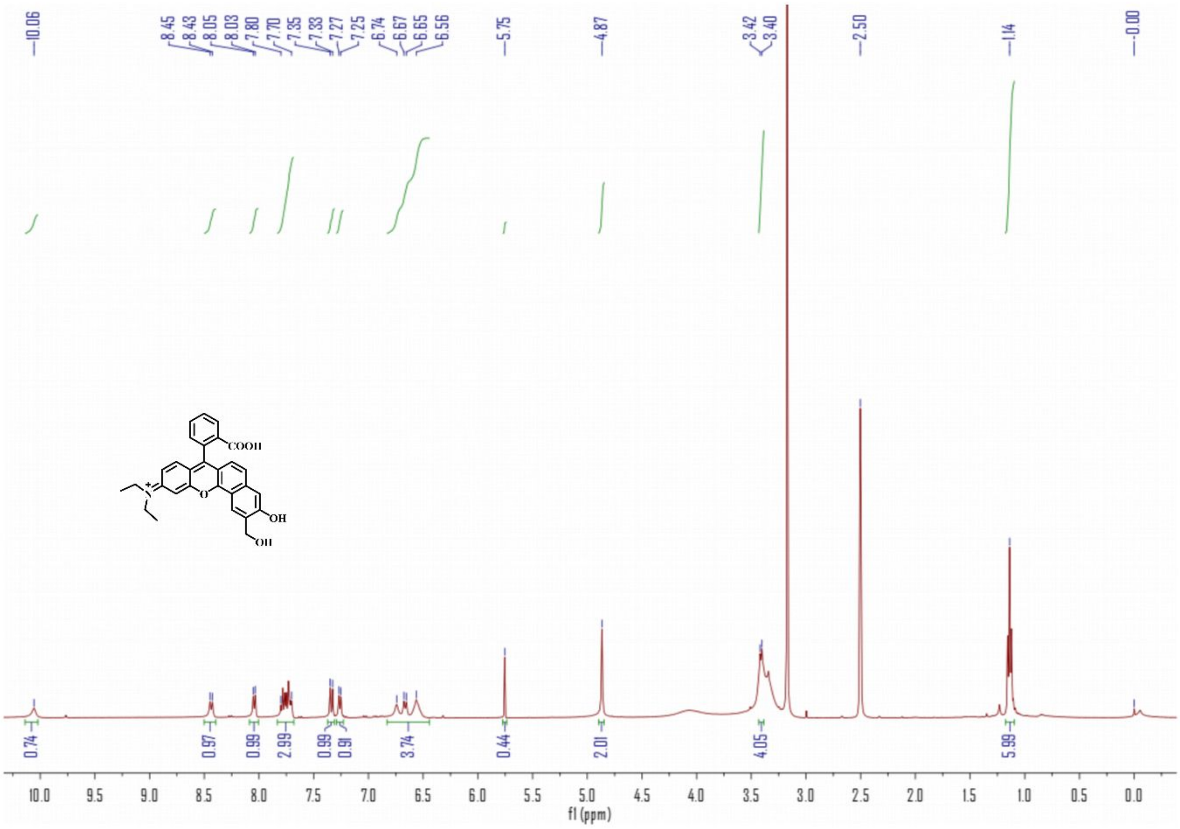

Figure S30 ^{1}H NMR (400 MHz, DMSO-d_6) of RB-FM-SA.

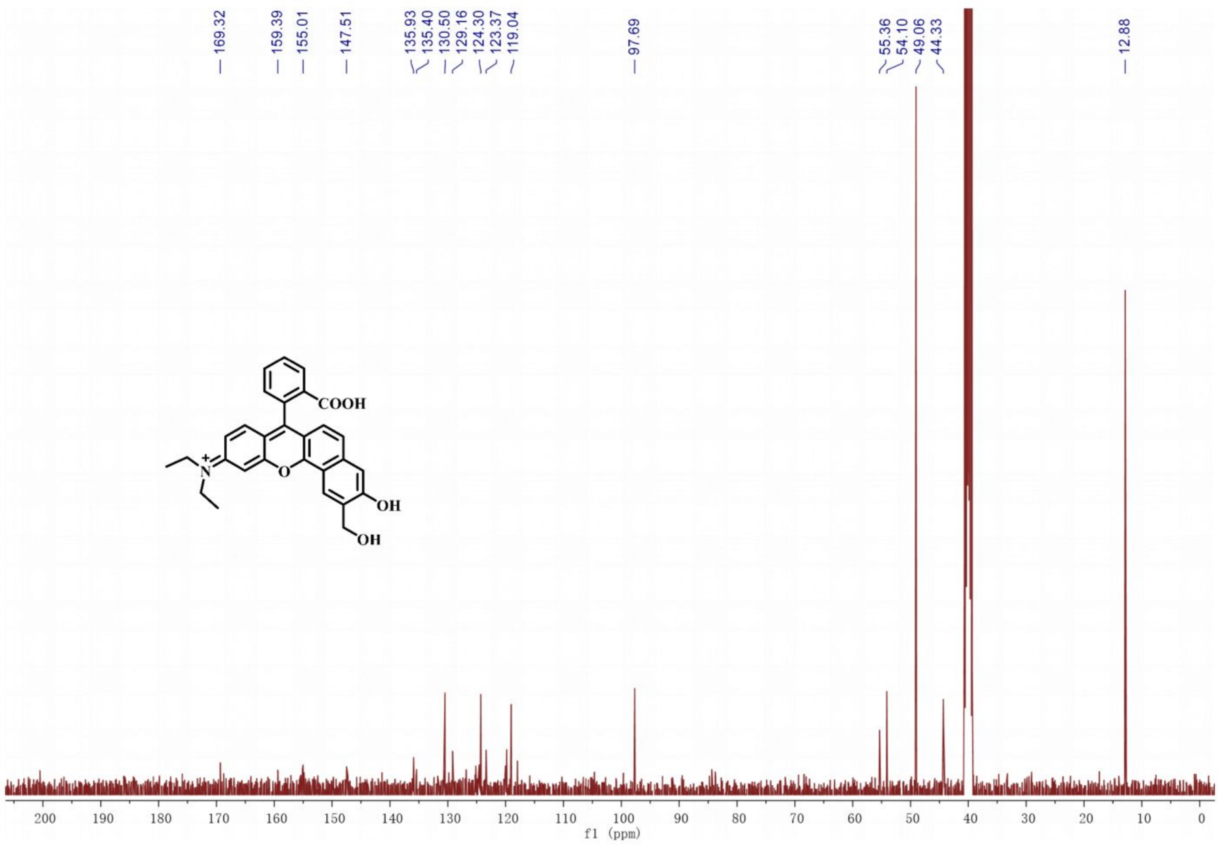

Figure S31 ^{13}C NMR (100 MHz, DMSO-d_6) of RB-FM-SA.

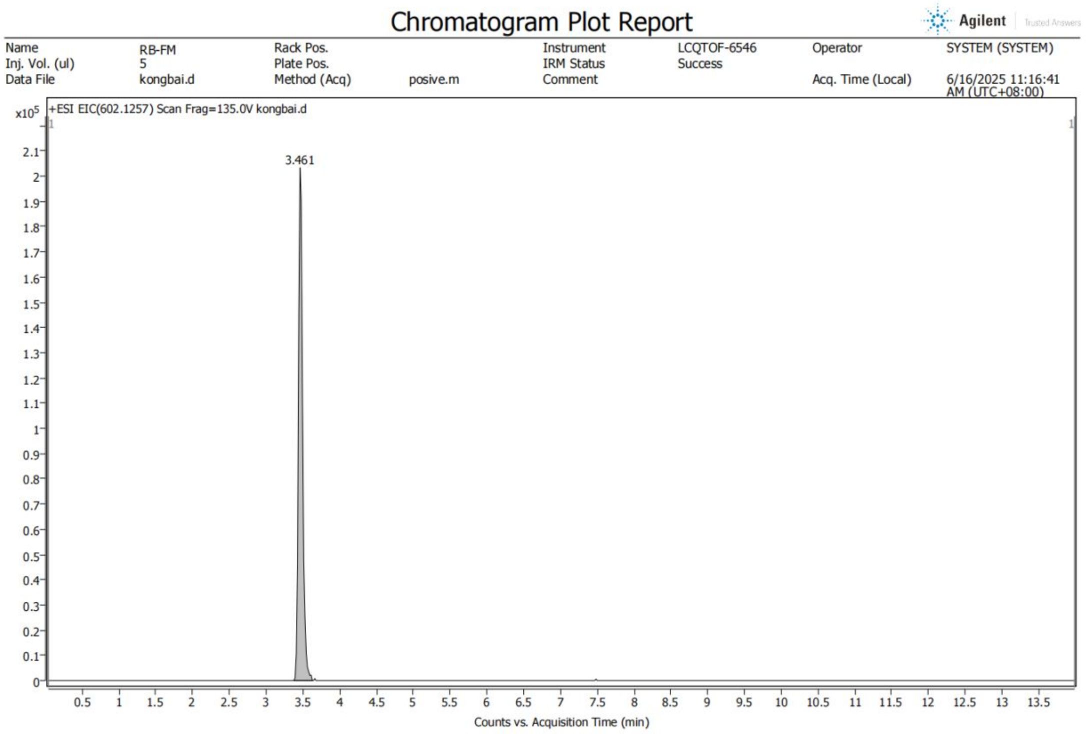

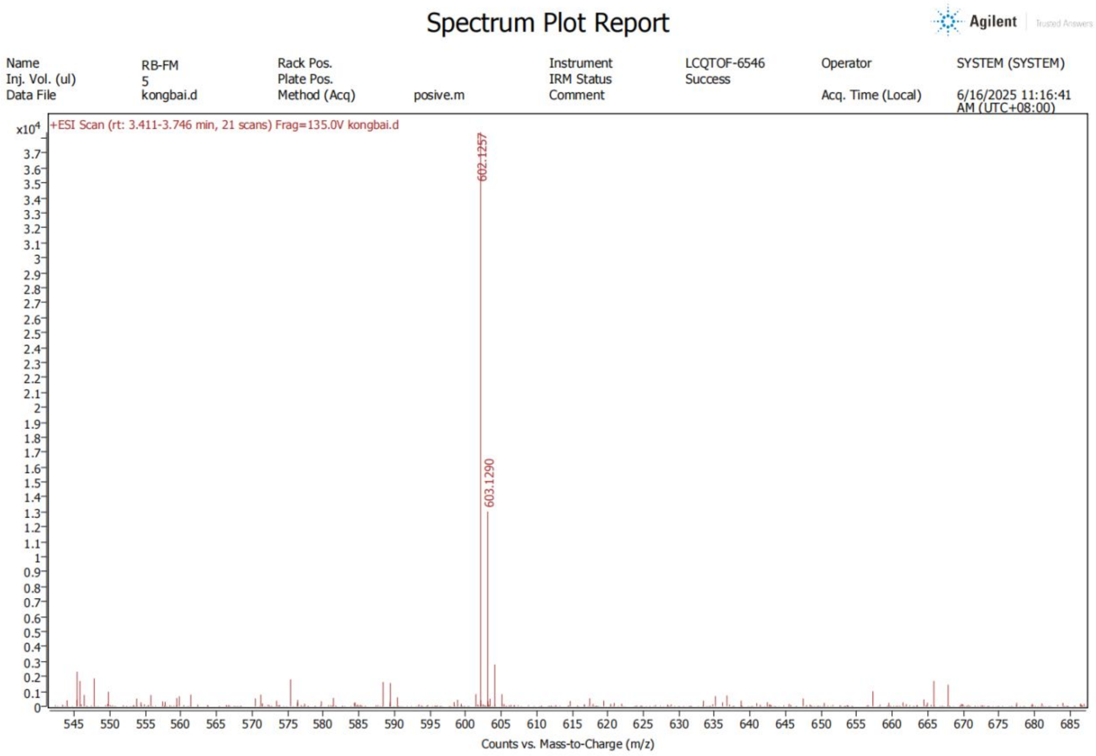

Figure S32. LC-MS of probe RB-FM.

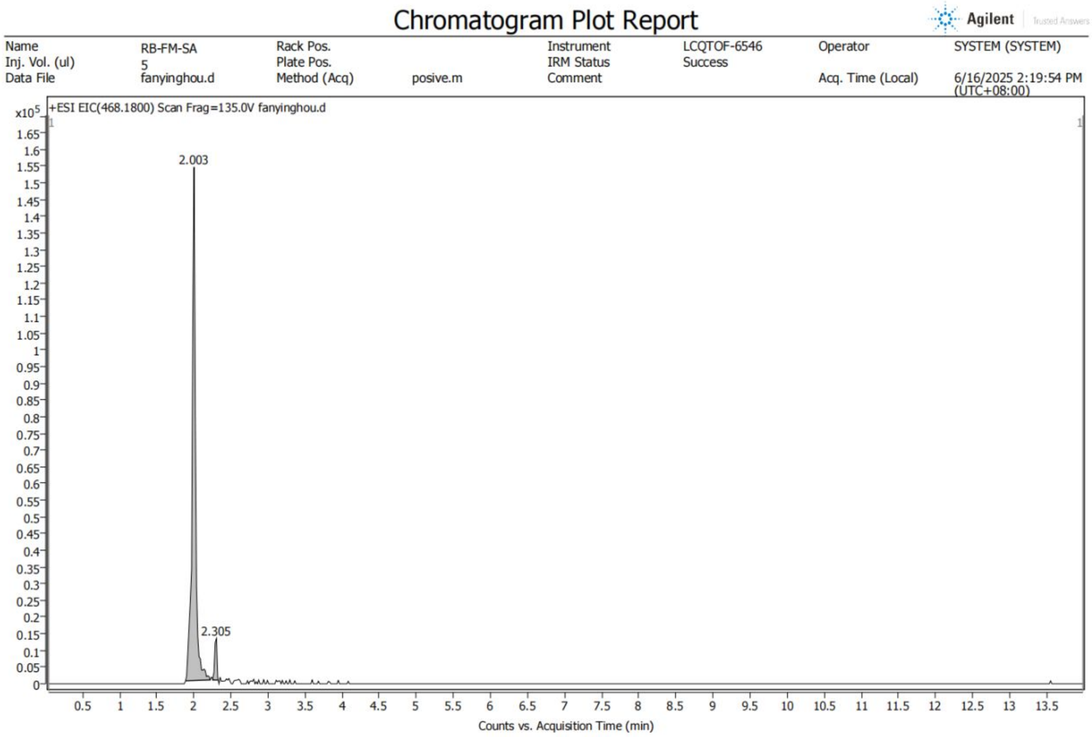

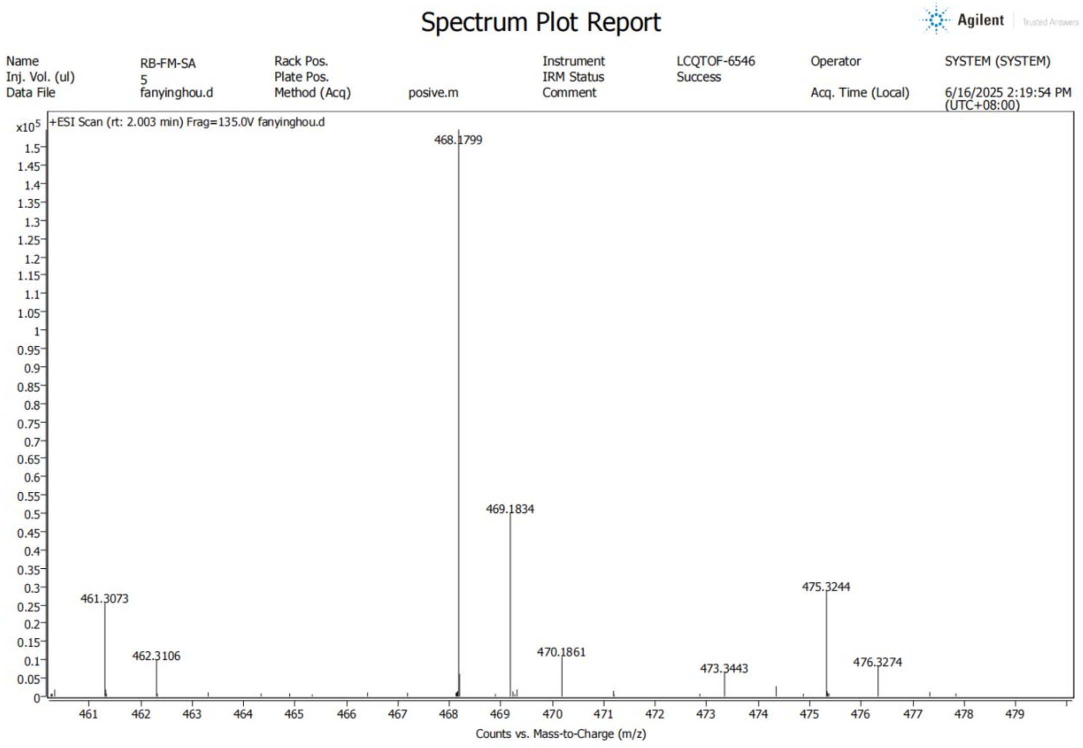

Figure S33. LC-MS of probe RB-FM + $O_2^{\bullet-}$, showing cleavage of RB-FM to the related phenol.

Table S1

Probe	Detection limit (nM)	Emission peak (nm)	Response time	Self-immobilizing ability	Ref.
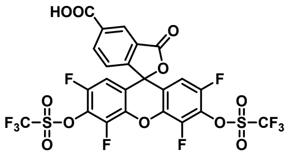	23	534	10 min	No	J. Am. Chem. Soc. 2015, 137, 6837-6843
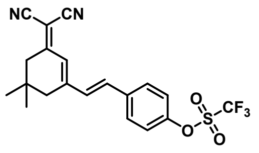	53	650	20 min	No	Anal. Chem. 2023, 95, 12240-12246
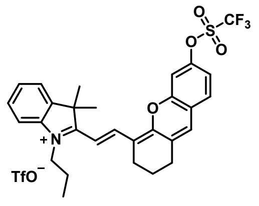	46.5	710	10 min	No	Chem. Sci. 2021, 12, 3921-3928
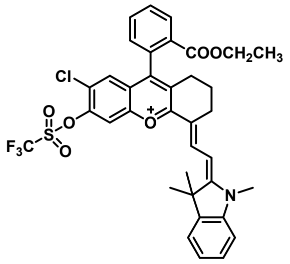	240	719	30 min	No	Chem. Sci. 2018, 9, 7606-7613
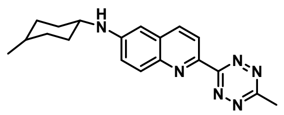	10	510	30 min	No	Nat. Commun. 14, 2023, 1401
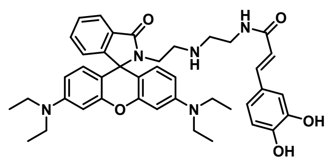	34	470	5 min	No	J. Am. Chem. Soc. 2023, 145, 19662-19675
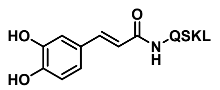	21.5	495	---	No	J. Am. Chem. Soc. 2020, 142, 20735-20743

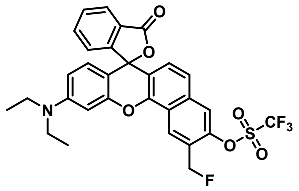	0.75	615	5 s	Yes	this work

References

1. Rong, X., et al. A long-wavelength mitochondria-targeted CO fluorescent probe for living cells and zebrafish imaging. *Anal. Methods* **16**, 442-448 (2024).
